# Environmental vibrios represent a source of antagonistic compounds that inhibit pathogenic *Vibrio cholerae* and *Vibrio parahaemolyticus* strains

**DOI:** 10.1002/mbo3.504

**Published:** 2017-08-30

**Authors:** David J. Burks, Stephen Norris, Kathryn M. Kauffman, Abigail Joy, Philip Arevalo, Rajeev K. Azad, Hans Wildschutte

**Affiliations:** ^1^ Department of Biological Sciences University of North Texas Denton Texas; ^2^ Department of Biological Sciences Bowling Green State University Bowling Green Ohio; ^3^ Department of Civil and Environmental Engineering Massachusetts Institute of Technology Cambridge Massachusetts; ^4^ Department of Mathematics University of North Texas Denton Texas

**Keywords:** antagonistic activity, antibiotics, biosynthetic gene clusters, pathogens, *Vibrio*

## Abstract

With the overuse of antibiotics, many pathogens including *Vibrio cholerae* and *Vibrio parahaemolyticus* have evolved multidrug resistance making treatment more difficult. While understanding the mechanisms that underlie pathogenesis is crucial, knowledge of bacterial interactions of *V. cholerae* and *V. parahaemolyticus* could provide insight to their susceptibility outside of the human host. Based on previous work showing competition among environmental strains, we predict that marine‐derived bacteria should inhibit *Vibrio* pathogens and may be a source of unique antibiotic compounds. We tested a collection of 3,456 environmental *Vibrio* isolates from diverse habitats against a panel of *V. cholerae* and *V. parahaemolyticus*, and identified 102 strains that inhibited the growth of these pathogens. Phylogenetic analysis revealed that 40 pathogen‐inhibiting strains were unique at the *hsp60* gene sequence while 62 of the isolates were identical suggesting clonal groups. Genomic comparisons of ten strains revealed diversity even between clonal isolates and were identified as being closely related to known *Vibrio crassostreae*,* Vibrio splendidus*, and *Vibrio tasmaniensis* strains. Further analysis revealed multiple biosynthetic gene clusters within all sequenced genomes that encoded secondary metabolites with potential antagonistic activity. Thus, environmental vibrios represent a source of compounds that inhibit *Vibrio* pathogens.

## 1 INTRODUCTION

The vast majority of vibrios, defined as bacteria belonging to the genus *Vibrio*, are harmless to humans. However, a few groups, in particular members of *Vibrio cholerae* and *Vibrio parahaemolyticus*, are well‐known pathogens and their virulence factors have been extensively studied for their roles in severe gastrointestinal diseases that cause cholera (Almagro‐Moreno, Pruss, & Taylor, 2015; Dziejman et al., 2002; Waldor & Mekalanos, 1996) and vibriosis (Hubbard et al., 2016; Makino et al., 2003) respectively. Cholera is a devastating disease that induces profuse watery diarrhea in which infected individuals can lose 10–20 L of fluid per day. If untreated, the mortality rate is 50%–60%, however, with immediate and proper action, recovery is greater than 99% (Sack, Sack, Nair, & Siddique, 2004). Most individuals can be treated by oral rehydration but in serve cases, antibiotic treatments are used to shorten the disease and reduce dehydration. Deoxycycline is typically the first option of antibiotic treatment. Unfortunately, many *Vibrio* pathogens have evolved resistance to this compound as well as other drugs used for treatment including cotrimoxazole, erythromycin, tetracycline, chloramphenicol, and furazolidone making novel antibiotic discovery a near future necessity for treating life threatening infections (Anand, Arora, Patwari, Agarwal, & Dewan, 1996; Carraro, Rivard, Ceccarelli, Colwell, & Burrus, 2016; Wang, Li, & Kan, 2016).

While populations of vibrios persist in many marine habitats (Thompson, et al., 2004, 2005), the natural reservoir for *V*. *cholerae* is uncertain. Cholera epidemics, caused by *ctx* encoding *V. cholerae* O1 and O139 types, tend to occur in a regular seasonal patterns (Alam et al., 2006; Mookerjee et al., 2014) and abundance has been correlated with warmer saline environments suggesting adaptation to certain habitats (Turner, Malayil, Guadagnoli, Cole, & Lipp, 2014). Other studies show that *V. cholerae* serotypes persist in marine waters worldwide, but in low abundance, and have been detected in many regions including South America, Australia, Sweden, and Italy that typically are void of cholera outbreaks (Collin & Rehnstam‐Holm, 2011; Islam et al., 2013; Schuster et al., 2011; Siboni, Balaraju, Carney, Labbate, & Seymour, 2016; Vezzulli et al., 2012). In the US, clinical cases of documented infections clearly demonstrate the presence of *Vibrio* pathogens in the coastal water column, and an estimated 80,000 individual infections per year occur from exposure to *V. cholerae* and *V. parahaemolyticus* via contaminated seafood and recreational beaches (Ralston, Kite‐Powell, & Beet, 2012; Scallan, Griffin, Angulo, Tauxe, & Hoekstra, 2011). From these studies, it is evident that pathogenic *V*. *cholerae* serotypes persists across marine environments. Particularly in regions where *V. cholerae* persists in low abundance, competition from other *Vibrio* groups may be strong. To that end, we provide the first investigation of population‐level competition against *Vibrio* pathogens as a means for natural compound discovery.

Previously, we showed that competition occurs among wild vibrios whereby distant relatives were antagonized and closely related strains were less susceptible to competitive interactions (Cordero, Wildschutte, et al., 2012). Given this demonstration, we hypothesized that environmental strains would yield the sought‐after inhibitory activity against devastating *Vibrio* pathogens. We thus screened a collection of 3,456 coastal marine vibrios against a panel of *V. cholerae* and *V. parahaemolyticus* clinical isolates. Using a plate assay to screen for antagonistic activity in one‐to‐one competitions, we identified 102 strains exhibiting strong inhibition against the panel. Thirty‐four distinct profiles of inhibition were observed, ranging from activity against a single to all eight pathogenic strains. The occurrence of multiple clonal groups, based on phylogenetic analysis using a housekeeping gene, from similar micro‐habitats suggested clonal expansions in the environment, however, genome sequence analyses revealed that even those strains were diverse. Biosynthetic gene clusters encoding secondary metabolites were identified within the sequenced genomes suggesting the production of inhibitory factors. Variable antagonistic profiles, ecotype analysis, and whole genome comparisons suggest these strains produce dissimilar factors and may be a source of novel inhibitory compounds.

## 2 RESULTS AND DISCUSSION

### 2.1 Rational for environmental strain selection

Given the expectation that environmental strains provide optimal sources for isolation of inhibitory compounds (Cordero, Wildschutte, et al., 2012), we chose to screen a large collection of previously isolated environmental marine vibrios (Kauffman, 2014) for activity against *Vibrio* pathogens. This environmental strain collection was sampled from the marine littoral zone of Nahant, MA, USA, on 3 days in late‐summer to early fall of 2010: August 10th (ordinal day 222), September 18th (ordinal day 261), and October 13th (ordinal day 286) when water temperatures were 13.8°C, 16.3°C, and 14.2°C respectively. A total of 3,456 strains were collected (Table 1), consisting of 1,152 strains from each of the 3 days and distributed evenly over particle‐associated (retained on 63 μm, 5 μm, or 1 μm filters) and free‐living isolates (passing through all filters and recovered on a 0.2 μm filter). Prior studies using a size‐fractionation approach showed that wild vibrios are highly diverse in both gene content (Polz, Hunt, Preheim, & Weinreich, 2006; Preheim et al., 2011; Thompson et al., 2005) and antagonistic interactions (Cordero, Wildschutte, et al., 2012), and could potentially yield diverse sources of inhibitory activity against pathogens. Moreover, this sampling strategy allows for bacterial collection from various resources ranging from larger phytoplankton (>63 fraction) to smaller habitats consisting of diverse marine microbes, detritus, and organic rich organic particles all which have been shown to support the presence of vibrios and may select for distinct antibiotic producing bacteria.

**Table 1:** Environmental marine *Vibrio* isolates used in the study and antagonistic activity

Habitat	Number of strains isolated (number of antagonistic strains) (Kauffman, 2014)			Total strains
	222 (August 10th)	261 (September 18th)	286 (October 13th)	
Free‐living	288 (5)	288 (30)	288 (14)	864 (49)
1 μm particle	288 (3)	288 (12)	288 (5)	864 (20)
5 μm particle	288 (6)	288 (3)	288 (4)	864 (13)
63 μm particle	288 (3)	288 (11)	288 (6)	864 (20)
Total strains	1,152 (17)	1,152 (56)	1,152 (29)	3,456 (102)

### 2.2 Pathogen‐inhibiting activity

**Figure 1:**
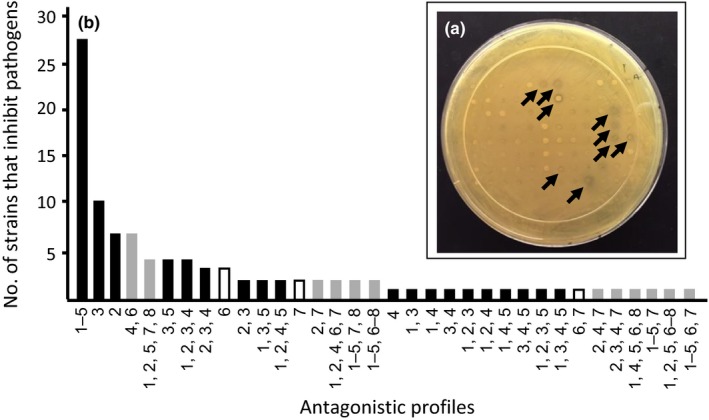
Inhibition assays of environmental strains against *Vibrio* pathogens reveal diverse profiles of antagonism. (a) A competition plate assay was used to determine inhibitory activity of environmental strains against a pathogen. Environmental strains were pin‐replicated onto a lawn of each pathogen, strains were cocultured for 18–24 hr at 23°C, and competitive interactions were defined by a zone of clearing of at least 1 mm. A photograph showing activity of environmental strains against the pathogen *V. cholerae* N16961 (indicated by the arrows). (b) Frequency of 34 antagonistic‐profiles among 102 pathogen‐inhibiting environmental isolates from 27,648 possible interactions. Antagonistic profiles are represented by unique combinations of numbers 1–8, representing the *V. cholerae* (1–5) and *V. parahaemolyticus* (6–8) strains listed below. Seventy‐one environmental isolates inhibited only *V. cholerae* (black bars) and six inhibited only *V. parahaemolyticus* strains (white bars). The remaining 25 strains inhibited members of both species (gray bars). Two environmental isolates were able to inhibit all pathogens (1–8) (VC, *V. cholerae*; VP, *V. parahaemolyticus*): 1, VC N19691; 2, VC O139; 3, VC MO101; 4, VC VO‐146; 5, VC VO‐258; 6, VP 954; 7, VP BB22OP; 8, VP EB101

In order to determine if pathogenic *V. cholera* and *V. parahaemolyticus* were susceptible to inhibition by marine‐derived isolates, all environmental strains (Table 1) were tested in competition assays against eight different human pathogens (Table 2). Five *V. cholerae* and three *V. parahaemolyticus* strains were utilized that differed by date of isolation, source of outbreak or infection, and serotype. Among the five *V. cholerae* strains, three were chosen that are involved in current and past cholera endemic and pandemic diseases [strain N16961 serotype O1 El Tor, strain O395 serotype O1 Classical, and strain MO10 serotype O139 El Tor (Dziejman et al., 2002; Mizunoe, Wai, Takade, & Yoshida, 1999)]; two other strains were chosen for being non‐O1 and non‐O139 serotypes yet caused gastrointestinal disease (Dalsgaard et al., 1999). The three *V. parahaemolyticus* strains were used to investigate activity against other non‐*V. cholerae* pathogens in order to determine if inhibition is species specific or exhibits a more broad range of activity (Fujino et al., 1965; Jensen et al., 2013). Pathogen susceptibility was determined using a high‐throughput plate assay in which strains were cocultured and subsequently screened for antagonistic activity (Figure 1A). We tested each of the 3,456 environmental isolates against the eight pathogens for a total of 27,648 individual interactions. Four hundred and five cases of inhibition from 102 isolates (~3.0% of the total collection) were identified that generated 34 distinct inhibition profiles (Figure 1B). Of the 102 isolates, two strains were able to inhibit all pathogens, 79 strains were able to inhibit multiple pathogens, and 23 isolates inhibited a single pathogen. A total of 77 strains showed species‐specific activity. For example 6/77 strains displayed inhibition against *V. parahaemolyticus* and 71/77 against *V. cholerae*, while 25 different environmental strains, including the aforementioned 12B9 (Cordero, Wildschutte, et al., 2012), exhibited interspecific activity and were able to inhibit both pathogenic *Vibrio* species (Figure 1B).

**Table 2:** *Vibrio* pathogens used in the study and observed antagonistic activity

Pathogen	Description	Reference	No. of environmental strains that antagonize
*V. cholerae* N16961	Serotype O1 El Tor	Dziejman et al. (2002); ATCC 39315	60
*V. cholerae* O395	Serotype O1	Dziejman et al. (2002); ATCC 39541	70
*V. cholerae* MO10	Serotype O139 El Tor	Mizunoe et al. (1999);	78
*V. cholerae* VO‐146	Serotype O10	Dalsgaard et al. (1999);	69
*V. cholerae* VO‐258	Serotype O8	Dalsgaard et al. (1999);	54
*V. parahaemolyticus* EB101	Shirasu food‐poisoning	Fujino et al. (1965); ATCC 17802	13
*V. parahaemolyticus* BB22OP	Strain LM5312	Jensen et al. (2013);	39
*V. parahaemolyticus* 954	Clinical isolate	Fujino et al. (1965) ATCC 49398	22
		Total antagonistic interactions	405

The antagonistic strains were isolated over spatiotemporal scales (Table 1). Of all 102 pathogen‐inhibiting strains, 17%, 55%, and 28% were isolated on Aug 10th, Sept 18th, and Oct 13th, respectively; and 48%, 20%, 12%, and 20% were isolated as free‐living from the water column or associated with 1 μm, 5 μm, and 63 μm size fractionated particles, respectively. No micro‐habitat or day of isolation was shown to significantly selected for antagonistic activity based on a Chi‐squared test. Of the total 102 antagonistic strains, 53 were isolated from three size‐fractionated suspended particles (Table 1) which represent nutrient rich carbon sources. The remaining 49 strains were free‐living isolates which may represent previously surface‐attached strains or strains that were removed from substrates during sample processing. Based on total *Vibrio* counts of 10^3^–10^4^ CFU/ml (Thompson et al., 2005) and that the 3% of the strains tested were inhibitory to pathogenic *Vibrio* strains, we estimated that 3×10^1^ to 3×10^2^ CFU/ml have the ability to outcompete *V. cholerae and V. parahaemolyticus* in this environment. This is a conservative estimate since our assays were performed on a single media type under conditions of constant temperature. These results show that natural isolates inhibit *Vibrio* pathogens.

### 2.3 Genotypic, ecological, and antagonistic diversity

**Figure 2:**
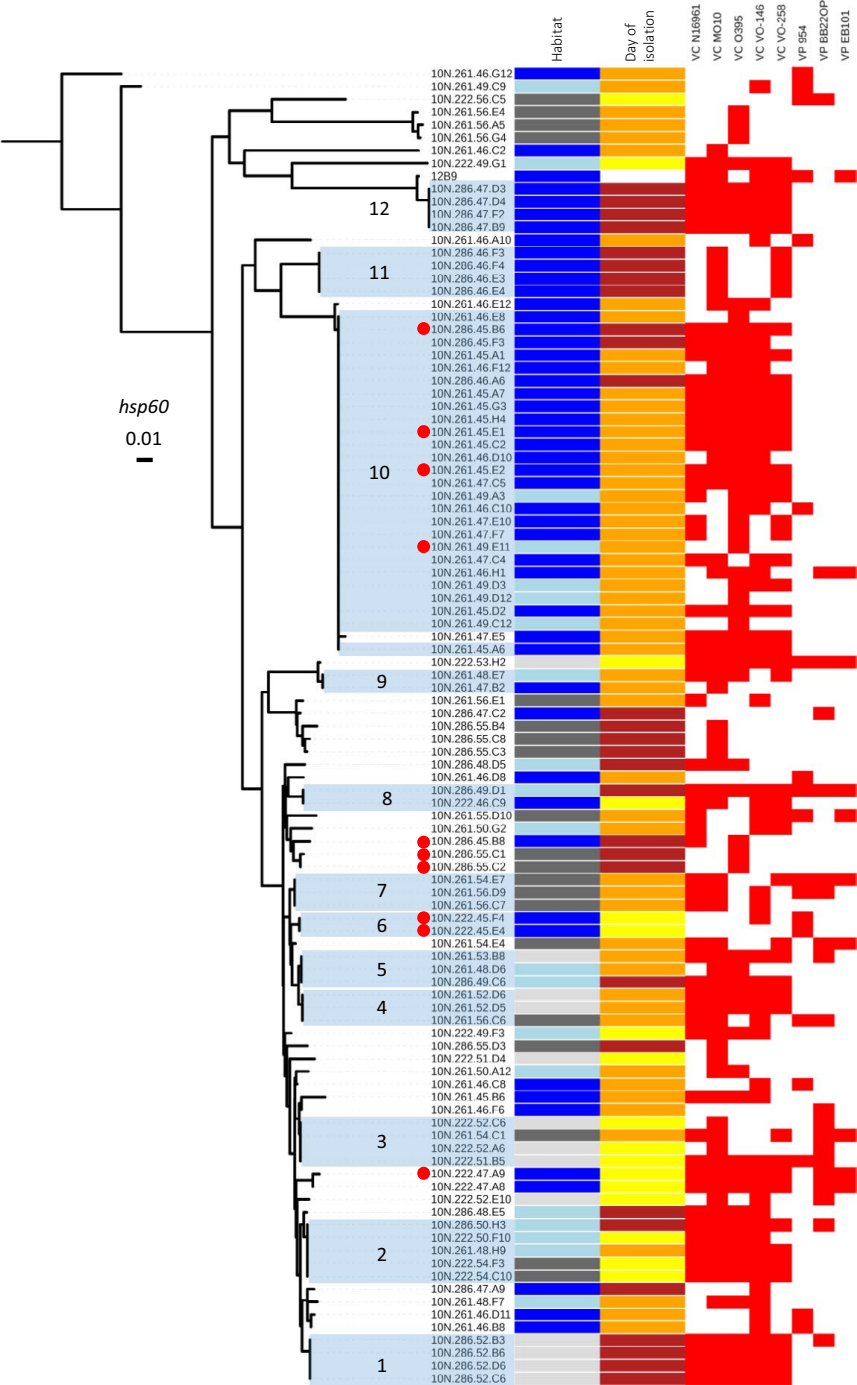
Phylogenetic tree of 102 antagonistic strains by neighbor‐joining analysis of the *hsp60* gene sequence overlaid with ecological and inhibitory data. Data are represented by color bars. The first column of bars represents habitat by particle size fractionation: dark gray, 63 μm; light gray, 5 μm; light blue 1 μm; and dark blue, free‐living. The second column represents ordinal day of isolation: yellow, 222; orange, 261; and maroon, 286. Antagonistic data are represented by the heat map. Inhibition is shown by a red square. VC and VP denote *V. cholerae* and *V. parahaemolyticus*, respectively, listed in Table 2. *hsp60* sequence type clonal groups 1–12 are shaded and identified. Strains whose genomes were sequenced are denoted by a red circle

To identify and determine the genetic relatedness of the environmental strains, a 453 bp internal region of the *hsp60* gene was amplified, sequenced, BLASTed against the NCBI nucleotide database, and used to create a neighbor‐joining phylogenetic tree (Figure 2). Nucleotide analysis confirmed that all strains were *Vibrio* isolates using a criteria with a 99%–100% query coverage and an *E*‐value of 0. The *hsp60* locus was chosen for phylogenetic analysis since it has previously been used to characterize the population‐level diversity of vibrios and provides a robust and more defined tree structure compared to the 16S rRNA gene (Cordero, Wildschutte, et al., 2012; Hunt et al., 2008; Kirchberger et al., 2016; Preheim et al., 2011). From phylogenetic results, we identified that 40 of the 102 strains were unique at *hsp60* nucleotide sequence. Ecotype diversity was observed by overlaying phylogeny with ecological (i.e., date of isolation and habitat) and inhibitory (i.e., killing profiles) data (Figure 2). Activity among these strains showed that patterns of inhibition are not predicted by ecotype suggesting different inhibitory mechanisms are expressed among wild isolates. Several strains had clear evidence of genetic diversity and differed in ecological content and antagonistic data. For example, strains 10N.222.47.A9 and 10N.286.45.B6 are distantly related based on phylogenetic clustering, and differ on the day of isolation and killing profile, suggesting each is a unique isolate. More closely related strains that differed based on habitat, such as 10N.261.56.E1 and 10N.286.47.C2, also were observed to exhibit diverse antagonistic activities, and even related strains (10N.222.45.E4 and 10N.222.45.F4) from similar habitats have different antagonistic phenotypes. Based on the collective results, we reason that these isolates produce distinct natural compounds that are capable of inhibiting *Vibrio* pathogens.

The other 62 vibrios were identical based on the *hsp60* gene (Figure 2, Groups 1–12) suggesting some isolates may represent clonal groups. Of these, 36 strains shared 100% nucleotide identity to at least one other of the 62 isolates while group 10 included 26 strains that were identical at *hsp60* locus; however, dissimilar ecological and killing profiles among some isolates suggest strain diversity. For instance, group 2 consists of five strains that are 100% identical based on the *hsp60* gene, yet the strains were isolated over three sampling days, from at least two defined habitats, and exhibited different killing profiles (Figure 2). Similarly, groups 4 and 5 consist of three strains each that differed in antagonistic activities and ecology. In group 10, only 9 of the 26 strains are identical based on antagonistic activity; the other 15 strains differ in killing profiles and ecological data. The diverse ecology and activity observed between strains in clonal groups suggest that these vibrios represent distinct isolates.

### 2.4 Genomic diversity among strains and species identification

To investigate if diversity occurs within clonal groups, we performed genome analysis on three groups of strains that consisted of closely related and clonal isolates to elucidate the differences and relatedness. We chose three strains that were closely related (10N.286.45.B8, 10N.286.55C1, and 10N.286.55.C2), isolated on the same day, and exhibited similar antagonistic profiles, and a more distantly related one (10N.222.47.A9) to confirm that the related strains (defined as Group X) differ in genome content (Figure 2, red circles). For clonal analyses, six strains from two different groups (Group 6: 10N.222.45.E4 and 10N.222.45.F4; Group 10: 10N.261.45.E1, 10N.261.45.E2, 10N.261.49.E11, and 10N.286.45.B6) were chosen to determine if these isolates represent identical strains as suggested by *hsp60* gene identity. Whole‐genome alignments of all strains were implemented using Mauve to obtain syntenic CDS and assess the genomic variability in each group.

**Figure 3:**
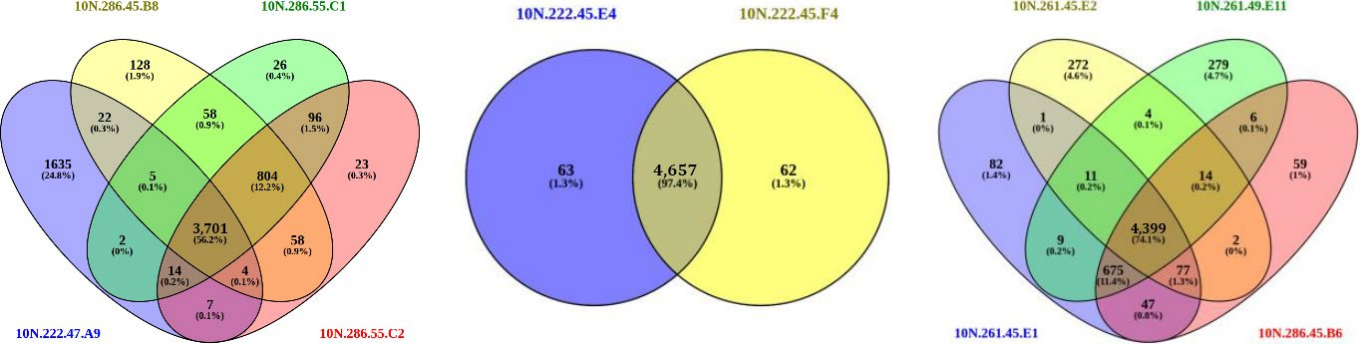
Genomic diversity among related strains. Venn diagrams showing the number and percentage of genes that are syntenic (or not) among the Group X (left), Group 6 (middle), and Group 10 (right) strains. Gene synteny was determined by Mauve; following the genome alignment, the overlapping gene models were identified as homologs within Mauve if they share at least 60% coverage and 70% identity at nucleotide level

Significant genomic variations were apparent in whole genome alignment of isolates within the three groups (Figure S1). Mauve comparison of the three closely related strains 10N.286.45.B8, 10N.286.55C1, and 10N.286.55.C2 with the more distant strain 10N.222.47.A9 revealed variations at the whole genome level (Figure S1A) and identified 3701 (56.2%) syntenic CDS between these based on default parameter setting of 60% coverage and 70% nucleotide identity (Figure 3). Most similarity was observed among the closely related isolates with ~3,500 identical genes found among the strains 10N.286.45.B8, 10N.286.55.C1, and 10N.286.55.C2, respectively (inclusive of exact gene copies in genomes), while only 293 genes in the more distantly related strain 10N.222.47.A9 were found to be identical to those in all three strains, based on protein BLAST (Camacho et al., 2009) (Table S1).

Genomic diversity was also observed among strains within the *hsp60* clonal groups. Strains 10N.222.54.E4 and 10N.222.54.F4 in clonal group 6 were identical based on ecotype but differ slightly in the competition profile suggesting strains are similar but nonclonal isolates (Figure 2). Mauve identified 4,657 (97.4%) syntenic CDS between these based on default parameter setting of 60% coverage and 70% identity (Figure 3, Figure S1B, and Table S2). Among Group 10 genomes, strains 10N.261.45.E1 and 10N.261.45.E2 are identical based on all data; strain 10N.286.45.B6 is identical to 10N.261.45.E1 and 10N.261.45.E2 based on habitat and killing data but differs on day of isolation; and strain 10N.261.49.E11 differs on habitat and antagonistic data (Figure 2). Mauve (Figure S1C) identified 4,399 (74.1%) syntenic CDS among the four isolates (Figure 3). Strain 10N.261.45.E2 was most variable as it encodes none of the 667 genes that are shared between genomes 10N.261.45.E1, 10N.261.49.E11, and 10N.286.45.B6 (Table S3). These analyses show that Groups 6 and 10 consist of strains that are nonclonal isolates. Based on the combined genomic, ecological, and killing data, we predict that many of the other 56 of 62 clonal strains with Groups 1–12 represent nonclonal variants.

Using the sequenced genomes of the environmental strains, we performed an average nucleotide identity (ANI) analysis against 1,713 *Vibrio* genomes currently available in the NCBI database to gain a better understanding of the population‐level diversity and species identification of the environmental strains. Pairwise ANI was computed using our ten strains and 12B09 against the *Vibrio* genome database using both local alignment algorithm BLAST (ANIb) and whole genome alignment algorithm MUMmer (ANIm). The best matches of the environmental strains are given in Table 3. The ANIb and ANIm results were similar with both methods identifying three *Vibrio* species, namely *Vibrio crassostreae*,* Vibrio splendidus*, and *Vibrio tasmaniensis*. The known *Vibrio ordalii* strain 12B09 (Cordero, Wildschutte, et al., 2012) was also analyzed and both ANIm and ANIb verified the species (Table 3). We also computed ANIs of the environmental strains aligned against strains from different genera but within the *Vibrionaceae* family including members from *Aliivibrio*,* Photobacterium*,* Enterovibrio,* and *Salinivibrio*. As expected, none of these ANI values was higher than those for *Vibrio*. The *Vibrio* groups identified, *V. crassostreae*,* V. splendidus*, and *V. tasmaniensis*, all have previously been isolated from coastal Massachusetts and support other studies that these populations persist in this environment and are associated with habitats of suspended particles and free‐living in the water column (Szabo et al., 2013; Takemura, Chien, & Polz, 2014). The results suggest that native populations, which persists across time and habitat, have the ability to inhibit *Vibrio* pathogens.

**Table 3:** Average nucleotide identity analysis using MUMmer (ANIm) and BLAST (ANIb) for each of the 10 environmental strains

Strain	ANIb/ANIm	Best matching species	Strain	NCBI genome accession
10N.222.45.E4	0.9170/0.9422	*splendidus*/*splendidus*	FF6/6195	GCA_000272325.2/GCA_000272345.2
10N.222.45.F4	0.9170/0.9236	*splendidus*/*splendidus*	FF6/13B01	GCA_000272325.2/GCA_001691275.1
10N.222.47.A9	0.9694/0.9706	*splendidus/splendidus*	13B01/13B01	GCA_001691275.1
10N.261.45.E1	0.9072/0.9132	*crassostreae*/*crassostreae*	Evh12/Evh12	GCA_001486525.1
10N.261.45.E2	0.9072/0.9132	*crassostreae/crassostreae*	Evh12/Evh12	GCA_001486525.1
10N.261.49.E11	0.9075/0.9135	*crassostreae/crassostreae*	Evh12/Evh12	GCA_001486525.1
10N.286.45.B6	0.9068/0.9132	*crassostreae/crassostreae*	Evh12/Evh12	GCA_001486525.1
10N.286.45.B8	0.9835/0.9847	*tasmaniensis*/*tasmaniensis*	5F79/5F‐79	GCA_000272425.1
10N.286.55.C1	0.9839/0.9848	*tasmaniensis*/*tasmaniensis*	5F79/5F‐79	GCA_000272425.1
10N.286.55.C2	0.9835/0.9848	*tasmaniensis*/*tasmaniensis*	5F79/5F‐79	GCA_000272425.1
12B09	0.9984/0.9994	*ordalii*/*ordalii*	FS144/FS‐144	GCA_000287115.2

### 2.5 Gene clusters encoding potential inhibitory factors

Antibiotic and Secondary Metabolite Analysis Shell (antiSMASH) was used to identify potential biosynthetic gene clusters (BGCs) that encode secondary metabolites which may be involved in toxigenic compound production (Weber et al., 2015). In the 10 strains, 4–6 BGCs were identified in each sequenced genome (Table 4). All strains encoded a predicted aryl‐polyene, bacteriocin, and polyunsaturated fatty acid (PUFA), and only 10N.222.45.E4 and 10N.222.45.F4 did not encode a predicted siderophore. In addition, all strains except 10N.286.45.B8 encoded either a putative non‐ribosomal peptide synthetase (NRPS) or a polyketide synthase (PKS); and 10N.261.45.E1, 10N.261.49.E11, and 10N.286.45.B6 were predicted to encode a homoserine lactone. BGC pairwise analysis showed that clusters encoding the same predicted product were similar in nucleotide identity among strains (87%–100%, Tables S4–S10). The BGCs encoding the putative NRPs and PKSs exhibited the most diversity, and the PKS in strain 10N.222.47.A9 was non‐homologous to all other BGCs. Bacteriocins (Carraturo, Raieta, Ottaviani, & Russo, 2006; Prasad, Morris, Hansen, Meaden, & Austin, 2005), NRPSs (Cordero, Wildschutte, et al., 2012), PKSs (Liaw et al., 2015), and siderophores (Chatterjee et al., 2017) are compounds that have been shown to exhibit antagonistic activity and these BGCs may contribute to the inhibition observed by the 10 strains; moreover, few genes in these cluster have similarity to other known loci (Table 4) suggesting these BGCs may encode novel products. Although these results identify putative regions encoding antagonistic factors on the chromosome, it is also possible that genes encoding inhibitory products originate on plasmids that would be undetected in our genomic analysis. A recent study by Xue et al. suggests that natural populations of marine vibrios host large numbers of plasmids and episomes, some of which encode plasmid‐like temperate phages (Xue et al., 2015) which may inhibit specific *Vibrio* strains when displaying a lytic lifecycle. Other studies show that *Vibrio* isolates carry plasmids that encode the production of siderophores (Alice, Lopez, & Crosa, 2005; Chen, Actis, Tolmasky, & Crosa, 1994; Tolmasky, Salinas, Actis, & Crosa, 1988) which are known to be involved in competition among bacteria (Chatterjee et al., 2017; Cordero, Ventouras, DeLong, & Polz, 2012). Thus, it is possible that the antagonistic activity we observed may originate from products encoded on the chromosome or a plasmid. Despite the location of these genes encoding antagonistic activity, these isolates potentially encode an arsenal of products that may be involved in antagonistic activity.

**Table 4:** Gene clusters and predicted products potentially involved in antagonistic activity

Strain	Clonal group	Predicted products (kb of gene cluster/no. ORFs in cluster/% of genes with known similarity)							
		Aryl‐polyene	Bacteriocin	Ectoine	Homoserinelactone	NRPS	PKS	PUFA	Siderophore
10N.222.45.E4	6	43.6/17/85	10.9/12/0	–	–	62.2/53/75	–	55.9/38/26	–
10N.222.45.F4	6	43.6/17/85	10.9/12/0	–	–	65.2/53/75	–	55.9/38/26	–
10N.261.45.E1	10	31.6/17/85	10.9/11/0	–	20.6/19/3	–	51.2/16/0	48.7/33/26	14.9/11/63
10N.261.45.E2	10	43.6/17/85	10.9/11/0	–	–	–	51.6/21/0	48.7/33/26	14.9/11/63
10N.261.49.E11	10	43.6/17/85	10.9/11/0	–	20.6/19/3	–	73.7/34/0	45.7/33/26	14.9/11/63
10N.286.45.B6	10	43.6/17/85	10.9/0	–	20.6/19/3	–	51.9/17/0	48.7/33/26	14.9/11/63
10N.222.47.A9	–	43.6/17/85	10.9/13/0	10.4/100	–	–	47.0/32/6	55.9/37/26	14.9/12/63
10N.286.45.B8	–	43.6/17/85	10.9/13/0	–	–	–	–	38.1/22/26	14.9/12/63
10N.286.55.C1	–	43.6/17/85	10.9/13/0	–	–	22.3/27/0	–	55.9/37/26	14.9/12/63
10N.286.55.C2	–	43.6/17/85	10.9/13/0	–	–	22.3/27/0	–	55.9/27/26	14.9/12/63

In summary, we show that environmental vibrios inhibit *V. cholerae* and *V. parahaemolyticus* pathogens. Marine‐derived strains exhibited diverse antagonistic profiles and were isolated across distinct habitats and time suggesting that inhibitory strains persist in the environment. Through genome sequence analysis, BGCs were identified that encode secondary metabolites which may contribute to the antagonistic phenotype. With the emergence of multidrug resistant pathogens, this information may be leveraged towards the application of selective pressure for the prevention or attenuation of *V. cholerae* outbreaks and novel antibiotic discovery.

## 3 MATERIALS AND METHODS

### 3.1 Environmental strains used

A collection of environmental marine vibrio strains previously isolated at Canoe Cove, Nahant, MA (Kauffman, 2014) was screened for activity against representative pathogens. This collection was comprised of free‐living and particle‐associated bacterial strains sampled from the marine littoral zone in Nahant, MA, on August 10th, September 18th, and October 13th of 2010 using a size‐fractionation approach (Hunt et al., 2008), briefly as follows. Bacteria associated with >63 μm particles were collected by passing seawater through a 63 μm phytoplankton net (Nitex Turtox Tow Net, Wild Co.), homogenizing the retentate in a tissue grinder (VWR), and then filtering onto 0.2 μm filters. Small‐particle‐associated bacteria and free‐living bacteria were recovered by passing 63 μm prefiltered seawater successively over 5 μm, 1 μm, and 0.2 μm polycarbonate filters. Filters for all size‐fractions were vortexed to release bacteria and dilution series of these suspensions were filtered onto 0.2 μm polyethersulfone filters for colony development by transfer onto *Vibrio* selective marine thiosulfate citrate bile salts sucrose (prepared according to manufacturer instruction with modification of 10 g NaCl per liter to 2% final w/v) (MTCBS Agar, Difco). A total of 96 colonies were selected from each replicate of each size fraction for each day and serially purified by restreaking first onto nonselective tryptic soy broth agar (TBS2, Difco TSB with 1.5% Difco Bacto Agar amended with 15 g NaCI to 2% w/v), then onto marine MTCBS, and finally again onto TBS2. Liquid cultures for frozen stocks were prepared by inoculation of a single colony into 2216 Marine Broth (Difco) and 200 μl of the resulting overnight culture was resuspend in a final of volume of 15% glycerol.

### 3.2 PCR amplification and phylogenetic analysis

Amplification of part of the *hsp60* gene was performed with all isolates using the H279 and H280 primers 5′‐GAATTCGAIIIIGCIGGIGAYGGIACIACIAC‐3′ and 5′‐CGCGGGATCCYKIYKITCICCRAAICCIGGIGCYTT‐3′ respectively (Goh et al., 1996). PCR amplification was carried out for 30 cycles of (92°C for 30 s, 40°C for 30 s, and 72°C for 60 s) following a 2 min initial extension at 92°C with a final extension of 5 min at 72°C. Individual isolates were cultured in TBS2 liquid broth and a 1 μl aliquot from each overnight culture subjected to PCR and sequencing. Sanger sequencing was performed using the H279 primer at University of Chicago Sequencing Center, Chicago, IL. The obtained *hsp60* gene sequences were aligned and a neighbor‐joining tree constructed using default parameters in CLC Main Workbench. Bootstrapping was performed with 100 replicates.

### 3.3 Competition assays

Strains were grown overnight for 20 hr prior to the time of assay in TSB2. To create a bacterial lawn, 50 μl of a pathogenic *Vibrio* culture was spread on TSB2 agar plates. Subsequently, 1 μl of the environmental strains, in stationary phase, were stamped onto the lawn from 96‐deep well plates using a replicator (Boekel 140500 Microplate Replicator). The assay was performed at 23°C, a temperature at which both the pathogen and environmental strain were able to grow. Zones of clearing were recorded between 18 and 24 hr after coculturing; 1 mm zone of inhibition around the stamped strains was scored positive for antagonistic activity. To test for false positive results, all inhibitory strains were selected and replicated at least three times against all pathogens.

### 3.4 Genome sequencing

Genomic DNA was extracted using the Wizard Genomic DNA Purification Kit (Promega). A DNA library was prepared for each of ten strains 10N.222.54.E4, 10N.222.54.F4, 10N.222.47.A9, 10N.286.45.B6, 10N.286.45.B8, 10N.286.55.C1, 10N.286.55.C2, 10N.261.45.E1, 10N.261.45.E2, 10N.261.49.E11 using the tagmentation‐based Illumina Nextera kit (Baym et al., 2015). Libraries were sequenced using 100 × 100 paired end reads using Illumina HiSeq Rapid technology (Whitehead Institute Genome Technology Core, Cambridge, MA). The genome was processed and assembled using a custom workflow in CLC Genomics Workbench 8.0.2. Specifically, data processing included removal of duplicate reads, adapter and quality trimming (0 ambiguous nucleotides allowed, quality limit 0.005), merger of overlapping reads, removal of short overlapped reads, and de novo assembly using default parameters. All genomes have been submitted to GenBank (Accession #s MCZZ00000000, MCYT00000000, MCWK00000000, MCWI00000000, MCSI00000000, MCSH00000000, MKKO00000000, MKKM00000000, MKKN00000000, MKKP00000000). For genome sequence review prior to release by GenBank, please contact co‐author Phil Arevalo at philip.a.arevalo@gmail.com.

### 3.5 Genome alignment and analysis

Multiple whole‐genome alignment was performed using Mauve (Darling, Mau, Blattner, & Perna, 2004) by invoking the progressiveMauve function with default parameters. For both Group 6 and Group 10 genomes, SNPs, orthologs, and alignment images were generated. Gap information was only exported for Group 6, as there were no detectable gaps within the contigs of the Group 10 genomes. Full alignment for all 10 analyzed *Vibrio* genomes was also performed with progressiveMauve. Exported ortholog lists were parsed with custom Python scripts, and visualized as Venn diagrams using the Venny 2.1 webtool (Oliveros, 2007).

### 3.6 Genic variations

Annotated, whole‐genome files for Group 6 and Group 10 isolates were parsed with the SeqIO function of Biopython (Cock et al., 2009). CDS were identified and translated to protein sequences. Genic variations, in terms of variations in gene products, in each group were examined via protein BLAST using the BLASTP command of the ncbi‐BLAST+ suite with an e‐value cut‐off of 10 (Camacho et al., 2009). The BLAST output was parsed with custom Python scripts to record variations observed in gene products in each group at different parameter settings (% query coverage and % identity). The BLASTP output was further analyzed with custom Python and Perl scripts to identify the ‘best‐match’ for a gene product within a group, identified by the lowest *E*‐value. In addition, SNPs were obtained for both Group 6 and Group 10 using Mauve.

### 3.7 Average nucleotide identity (ANI) analysis

To identify the known vibrio species that are represented in our collection of ten environmental strains, we downloaded all 1,713 *Vibrio* genome fasta nucleotide (.fna) files available in the NCBI FTP repository (ftp://ftp.ncbi.nlm.nih.gov/) and then computed average nucleotide identity (ANI) for each environmental strain with the known vibrios by aligning against each vibrio genome in our custom database. ANI alignments using both BLAST (ANIb) and MUMmer (ANIm) were performed by the Pyani 0.2.1 Python3 module (https://github.com/widdowquinn/pyani), and a custom Python3 wrapper that allowed the genomes of the environmental strains to be sequentially tested against each database genome. While MUMmer‐based ANI calculation uses whole‐genome alignments obtained through NUCmer (Kurtz et al., 2004), BLAST‐based ANI calculation entails fragmentation of each genome into 1020 bp sequences that are then aligned using the blastn function of the ncbi‐blast+ package (Camacho et al., 2009). ANI calculations provided by Pyani are interpreted according to previously established guidelines for species definition (Richter & Rossello‐Mora, 2009).
