# Supplementary material for: Environmental vibrios represent a source of antagonistic compounds that inhibit pathogenic Vibrio cholerae and Vibrio parahaemolyticus strains
Source: Microbiologyopen. 2017 Aug 30;6(5):e00504. doi: 10.1002/mbo3.504 (PMC5635165; doi:10.1002/mbo3.504)
Supplement: Supplementary file 1 [file MBO3-6-na-s001.pdf]

A

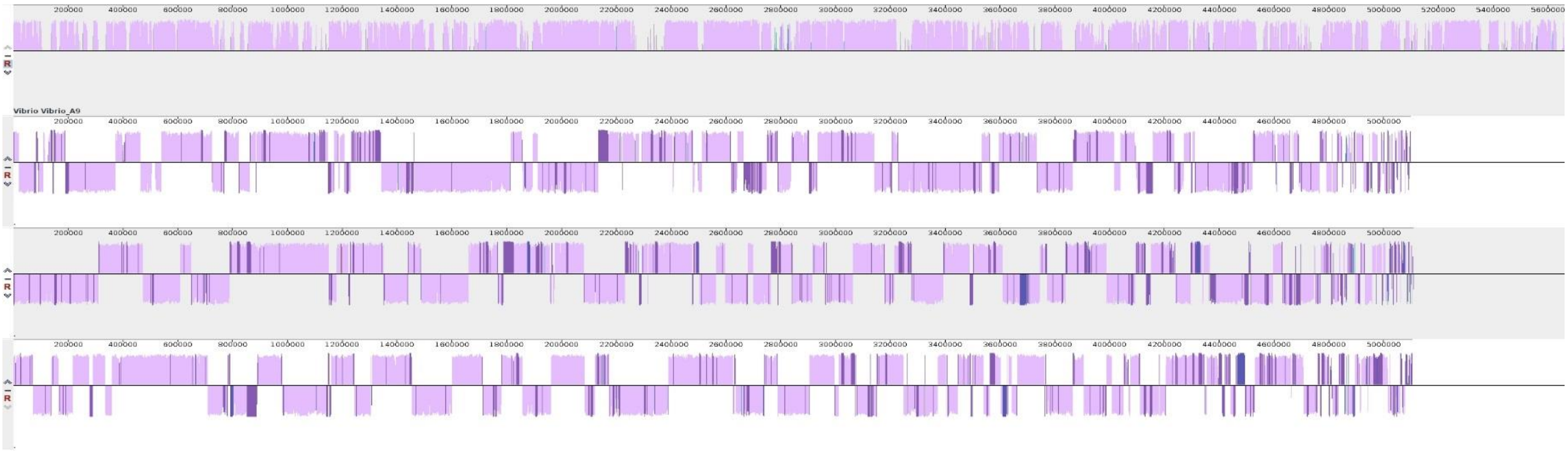

B

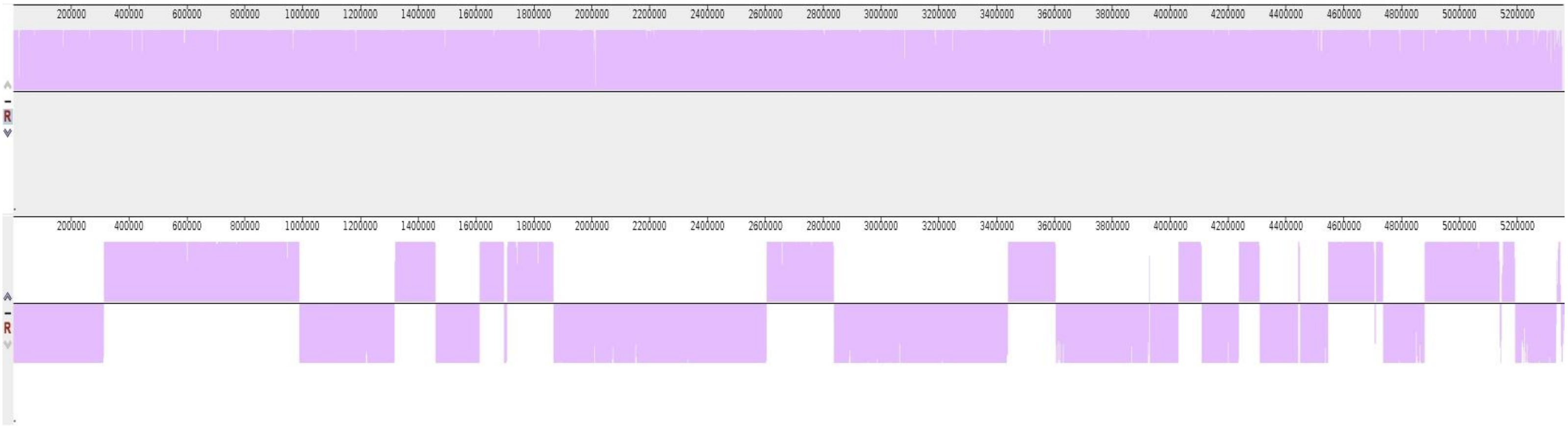

C

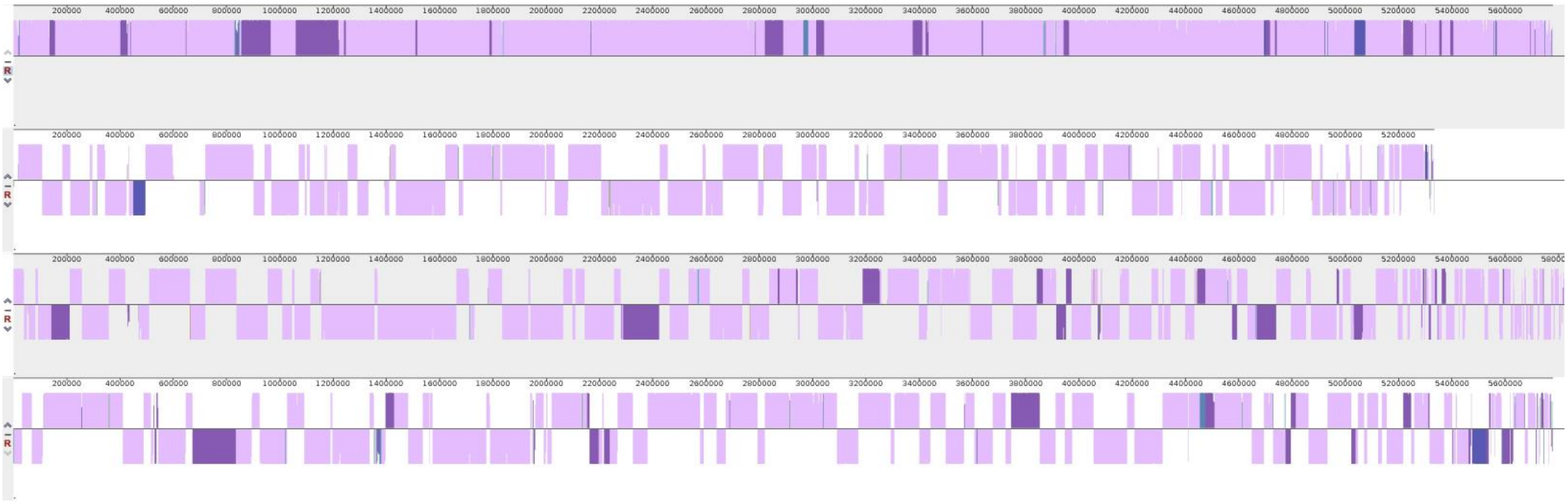

Supplemental Figure 1. Mauve whole-genome alignments showing genomic variations. Alignments were performed among A) Group X (top to bottom: 222.47.A9, 286.45.B8, 286.55.C1, and 286.55.C2), B) Group 6 (top to bottom: 222.45.E4, 222.45.F4), and C) Group 10 (261.45.E1, 261.45.E2, 261.49.E11, and 286.45.B6) genomes. Regions of alignment shared amongst all genomes is represented in light purple. Regions shared among only a subset of the genomes is represented in different colors.
