# Supplementary material for: Environmental vibrios represent a source of antagonistic compounds that inhibit pathogenic Vibrio cholerae and Vibrio parahaemolyticus strains
Source: Microbiologyopen. 2017 Aug 30;6(5):e00504. doi: 10.1002/mbo3.504 (PMC5635165; doi:10.1002/mbo3.504)
Supplement: Supplementary file 2 [file MBO3-6-na-s002.docx]

Supplementary Table 1. Genic variations observed amongst the Group X genomes. Identical and variant gene products were identified by comparison of translated gene sequences using BLASTP (at stringency of 100% coverage and 100% identity at amino-acid level).

|  | 10N.222.47.A9 | 10N.286.45.B8 | 10N.286.55.C1 | 10N.286.55.C2 |
| --- | --- | --- | --- | --- |
| A9 Only | 4889 | 0 | 0 | 0 |
| B8 Only | 0 | 754 | 0 | 0 |
| C1 Only | 0 | 0 | 51 | 0 |
| C2 Only | 0 | 0 | 0 | 85 |
| A9, C1, and C2 | 12 | 0 | 12 | 12 |
| A9 and C1 | 1 | 0 | 2 | 0 |
| B8 and C1 | 0 | 32 | 32 | 0 |
| B8, C1, and C2 | 0 | 3564 | 3567 | 3563 |
| A9, B8, C1, and C2 | 293 | 296 | 296 | 295 |
| C1 and C2 | 0 | 0 | 720 | 717 |
| B8 and C2 | 0 | 10 | 0 | 10 |
| A9 and B8 | 15 | 15 | 0 | 0 |

Supplementary Table 2. Number of genes in each of the two genomes of Group 6 with % coverage or % identity in “X to Y” range in alignment with their best blastp hit among the other three genomes.

|  | 10N.222.45.E4 | | 10N.222.45.F4 | |
| --- | --- | --- | --- | --- |
| X to Y (%) | Number of genes with “X to Y” % coverage in blastp alignment | Number of genes with “X to Y” % identity in blastp alignment | Number of genes with “X to Y” % coverage in blastp alignment | Number of genes with “X to Y” % identity in blastp alignment |
| 0 to 10 | 10 | 1 | 16 | 4 |
| 10 to 20 | 4 | 0 | 8 | 0 |
| 20 to 30 | 3 | 2 | 5 | 0 |
| 30 to 40 | 0 | 8 | 4 | 9 |
| 40 to 50 | 2 | 7 | 3 | 6 |
| 50 to 60 | 12 | 23 | 6 | 24 |
| 60 to 70 | 3 | 13 | 4 | 14 |
| 70 to 80 | 2 | 8 | 1 | 10 |
| 80 to 90 | 4 | 7 | 3 | 5 |
| 90 to 100 | 4679 | 4650 | 4670 | 4648 |

Supplementary Table 3. Genic variations observed amongst the Group 10 genomes. Identical and variant gene products were identified by comparison of translated gene sequences using BLASTP (at stringency of 100% coverage and 100% identity at amino-acid level).

| **Gene Distributions** | **E1** | **E2** | **E11** | **B6** |
| --- | --- | --- | --- | --- |
| E1 Only | 59 | 0 | 0 | 0 |
| E2 Only | 0 | 248 | 0 | 0 |
| E11 Only | 0 | 0 | 240 | 0 |
| B6 Only | 0 | 0 | 0 | 39 |
| E11 and B6 Only | 0 | 0 | 14 | 13 |
| E2 and B6 Only | 0 | 7 | 0 | 7 |
| E2, E11, and B6 Only | 0 | 17 | 17 | 15 |
| E2 and E11 Only | 0 | 13 | 13 | 0 |
| E1 and E2 Only | 3 | 3 | 0 | 0 |
| E1 and E11 Only | 7 | 0 | 7 | 0 |
| E1, E2, and E11 Only | 8 | 8 | 8 | 0 |
| E1 and B6 Only | 57 | 0 | 0 | 57 |
| E1, E2 and B6 Only | 72 | 74 | 0 | 73 |
| E1, E11, and B6 Only | 667 | 0 | 667 | 667 |
| All Four Genomes | 4428 | 4410 | 4431 | 4408 |

| Supplemental Table 4. BGC nucleotide pairwise analysis of the predicted arylpolyene product. | | | | | | | | | | |
| --- | --- | --- | --- | --- | --- | --- | --- | --- | --- | --- |
|  | 222.45.E4 | 222.45.F4 | 222.47.A9 | 261.45.E1 | 261.45.E2 | 261.49.E11 | 286.45.B6 | 286.45.B8 | 286.55.C1 | 286.55.C2 |
| 222.45.E4 | - | 100 | 91 | 88 | 88 | 88 | 88 | 92 | 92 | 92 |
| 222.45.F4 | 100 | - | 91 | 88 | 88 | 88 | 88 | 92 | 92 | 92 |
| 222.47.A9 | 91 | 91 | - | 89 | 89 | 89 | 89 | 95 | 95 | 95 |
| 261.45.E1 | 88 | 88 | 89 | - | 100 | 100 | 100 | 88 | 88 | 88 |
| 261.45.E2 | 88 | 88 | 89 | 100 | - | 100 | 100 | 88 | 88 | 88 |
| 261.49.E11 | 88 | 88 | 89 | 100 | 100 | - | 100 | 88 | 88 | 88 |
| 286.45.B6 | 88 | 88 | 89 | 100 | 100 | 100 | - | 88 | 88 | 88 |
| 286.45.B8 | 92 | 92 | 95 | 88 | 88 | 88 | 88 | - | 99 | 99 |
| 286.55.C1 | 92 | 92 | 95 | 88 | 88 | 88 | 88 | 99 | - | 100 |
| 286.55.C2 | 92 | 92 | 95 | 88 | 88 | 88 | 88 | 99 | 100 | - |

| Supplemental Table 5. BGC nucleotide pairwise analysis of the predicted bacteriocin. | | | | | | | | | | |
| --- | --- | --- | --- | --- | --- | --- | --- | --- | --- | --- |
|  | 222.45.E4 | 222.45.F4 | 222.47.A9 | 261.45.E1 | 261.45.E2 | 261.49.E11 | 286.45.B6 | 286.45.B8 | 286.55.C1 | 286.55.C2 |
| 222.45.E4 | - | 100 | 94 | 91 | 91 | 91 | 91 | 95 | 95 | 95 |
| 222.45.F4 | 100 | - | 94 | 91 | 91 | 91 | 91 | 95 | 95 | 95 |
| 222.47.A9 | 94 | 94 | - | 90 | 90 | 90 | 90 | 93 | 93 | 93 |
| 261.45.E1 | 91 | 91 | 90 | - | 100 | 100 | 100 | 91 | 91 | 91 |
| 261.45.E2 | 91 | 91 | 90 | 100 | - | 100 | 100 | 91 | 91 | 91 |
| 261.49.E11 | 91 | 91 | 90 | 100 | 100 | - | 100 | 91 | 91 | 91 |
| 286.45.B6 | 91 | 91 | 90 | 100 | 100 | 100 | - | 91 | 91 | 91 |
| 286.45.B8 | 95 | 95 | 93 | 91 | 91 | 91 | 91 | - | 99 | 99 |
| 286.55.C1 | 95 | 95 | 93 | 91 | 91 | 91 | 91 | 99 | - | 100 |
| 286.55.C2 | 95 | 95 | 93 | 91 | 91 | 91 | 91 | 99 | 100 | - |

| Supplemental Table 6. BGC nucleotide pairwise analysis of the predicted homoserine lactone product. | | | |
| --- | --- | --- | --- |
|  | 261.45.E1 | 261.49.E11 | 286.45.B6 |
| 261.45.E1 | - | 100 | 100 |
| 261.49.E11 | 100 | - | 100 |
| 286.45.B6 | 100 | 100 | - |

| Supplemental Table 7. BGC nucleotide pairwise analysis of the predicted NRPS product. | | | | |
| --- | --- | --- | --- | --- |
|  | 222.45.E4 | 222.45.F4 | 286.55.C1 | 286.55.C2 |
| 222.45.E4 | - | 100 | 87 | 87 |
| 222.45.F4 | 100 | - | 87 | 87 |
| 286.55.C1 | 87 | 87 | - | 100 |
| 286.55.C2 | 87 | 87 | 100 | - |

| Supplemental Table 8. BGC nucleotide pairwise analysis of the predicted PKS product. | | | | | |
| --- | --- | --- | --- | --- | --- |
|  | 222.47.A9 | 261.45.E1 | 261.45.E2 | 261.49.E11 | 286.45.B6 |
| 222.47.A9 | - | 0 | 0 | 0 | 0 |
| 261.45.E1 | 0 | - | 100 | 99 | 99 |
| 261.45.E2 | 0 | 100 | - | 100 | 100 |
| 261.49.E11 | 0 | 99 | 100 | - | 99 |
| 286.45.B6 | 0 | 99 | 100 | 99 | - |

| Supplemental Table 9. BGC nucleotide pairwise analysis of the predicted PUFA product. | | | | | | | | | | |
| --- | --- | --- | --- | --- | --- | --- | --- | --- | --- | --- |
|  | 222.45.E4 | 222.45.F4 | 222.47.A9 | 261.45.E1 | 261.45.E2 | 261.49.E11 | 286.45.B6 | 286.45.B8 | 286.55.C1 | 286.55.C2 |
| 222.45.E4 | - | 100 | 94 | 91 | 91 | 91 | 91 | 94 | 94 | 94 |
| 222.45.F4 | 100 | - | 94 | 91 | 91 | 91 | 91 | 94 | 94 | 94 |
| 222.47.A9 | 94 | 94 | - | 91 | 91 | 91 | 91 | 92 | 93 | 93 |
| 261.45.E1 | 91 | 91 | 91 | - | 100 | 100 | 100 | 91 | 91 | 91 |
| 261.45.E2 | 91 | 91 | 91 | 100 | - | 100 | 100 | 91 | 91 | 91 |
| 261.49.E11 | 91 | 91 | 91 | 100 | 100 | - | 100 | 91 | 91 | 91 |
| 286.45.B6 | 91 | 91 | 91 | 100 | 100 | 100 | - | 91 | 91 | 91 |
| 286.45.B8 | 94 | 94 | 92 | 91 | 91 | 91 | 91 | - | 99 | 99 |
| 286.55.C1 | 94 | 94 | 93 | 91 | 91 | 91 | 91 | 99 | - | 100 |
| 286.55.C2 | 94 | 94 | 93 | 91 | 91 | 91 | 91 | 99 | 100 | - |

| Supplemental Table 10. BGC nucleotide pairwise analysis of the predicted siderophore. | | | | | | | | |
| --- | --- | --- | --- | --- | --- | --- | --- | --- |
|  | 222.47.A9 | 261.45.E1 | 261.45.E2 | 261.49.E11 | 286.45.B6 | 286.45.B8 | 286.55.C1 | 286.55.C2 |
| 222.47.A9 | - | 92 | 92 | 92 | 92 | 91 | 91 | 91 |
| 261.45.E1 | 92 | - | 100 | 100 | 100 | 89 | 89 | 89 |
| 261.45.E2 | 92 | 100 | - | 100 | 100 | 89 | 89 | 89 |
| 261.49.E11 | 92 | 100 | 100 | - | 100 | 89 | 89 | 89 |
| 286.45.B6 | 92 | 100 | 100 | 100 | - | 89 | 89 | 89 |
| 286.45.B8 | 91 | 89 | 89 | 89 | 89 | - | 99 | 99 |
| 286.55.C1 | 91 | 89 | 89 | 89 | 89 | 99 | - | 100 |
| 286.55.C2 | 91 | 89 | 89 | 89 | 89 | 99 | 100 | - |

Partial hsp60 gene sequences

>12B9

TTCAAGTGCAGGAAGCAGTTCGCGAATGTTAGAAATTTTCTTATCAACCAATAGGATGAATGGGCTATCTAGATCAACACTGCCCGCTTCTTGGTTATTAATGAAGTAAGGAGATAGGTAACCACGATCAAACTGCATACCTTCCACTACATCGAGTTCATCTTGCAGTGCTTGACCTTCTTCAACAGTGATAACGCCATCTCGGCCCACTTTTTCCATCGCTTCAGCAATGATGTTACCCACGCTTGAATCCGAGTTCGCAGAAATAGTACCCACTTGCGCAATCGCTTTAGTGTCTGAACAAGGTACTGAAAGCTCTTTCAACGCTTCAACCGCCGCCACGACAGCTTTGTCGATGCCACGCTTAAGATCCATTGGGTTCATACCCGCTGCTACAGCTTTCAAGCCTTCAGTGATGATCGCTTGCGCTAATACCGTTGCGGTCGTCGTCCC

>10N.222.45.E4 TTCTAGAGTCGGAAGAAGTTCACGGATGTTCGATACTTTCTTGTCGATAAGAAGGATGAATGGGCTTTCTAGATCAACAGAACCCGCTTCTTGGTTGTTGATGAAGTAAGGAGATAGGTAACCGCGGTCGAACTGCATACCTTCAACTACGTCTAGCTCGTCTTGTAGAGCCTGACCTTCTTCAACTGTGATTACGCCATCACGACCTACTTTTTCCATCGCTTCAGCAATGATGTTACCTACTGTCGAATCAGAGTTCGCAGAGATAGTACCTACTTGAGCGATAGCTTTCGTGTCTGAACAAGGAACAGATAGGTTCTTCAGCTCTTCAACAGCCGCGATAACCGCTTTGTCGATGCCGCGCTTAAGATCCATTGGGTTCATGCCAGCAGCAACCGCTTTTAGGCCTTCAGCGATAATAGACTGAGCCAATACTGTTGCTGTCGTCGTCCC

>10N.222.45.F4 TTCTAGAGTCGGAAGAAGTTCACGGATGTTCGATACTTTCTTGTCGATAAGAAGGATGAATGGGCTTTCTAGATCAACAGAACCCGCTTCTTGGTTGTTGATGAAGTAAGGAGATAGGTAACCGCGGTCGAACTGCATACCTTCAACTACGTCTAGCTCGTCTTGTAGAGCCTGACCTTCTTCAACTGTGATTACGCCATCACGACCTACTTTTTCCATCGCTTCAGCAATGATGTTACCTACTGTCGAATCAGAGTTCGCAGAGATAGTACCTACTTGAGCGATAGCTTTCGTGTCTGAACAAGGAACAGATAGGTTCTTCAGCTCTTCAACAGCCGCGATAACCGCTTTGTCGATGCCGCGCTTAAGATCCATTGGGTTCATGCCAGCAGCAACCGCTTTTAGGCCTTCAGCGATAATAGACTGAGCCAATACTGTTGCTGTCGTCGTCCC

>10N.222.46.C9 TTCTAGAGTCGGAAGAAGTTCACGGATATTCGAAACTTTCTTGTCGATAAGAAGAATGAATGGGCTTTCTAAATCAACAGAACCTGCTTCTTGGTTGTTGATGAAGTAAGGAGACAGGTAACCGCGGTCGAACTGCATACCTTCAACTACGTCTAGCTCGTCTTGCAGAGCCTGACCTTCTTCAACCGTGATTACGCCATCACGACCTACTTTTTCCATCGCTTCAGCAATGATGTTACCTACTGTCGAATCAGAGTTCGCAGAGATAGTACCTACTTGCGCGATAGCTTTCGTGTCTGAACAAGGAACAGAAAGGTTCTTCAACTCTTCAACAGCCGCGATAACCGCTTTGTCGATGCCGCGCTTAAGATCCATTGGGTTCATGCCAGCAGCAACCGCTTTTAGGCCTTCTGCGATAATAGACTGAGCCAATACTGTTGCTGTCGTCGTCCC

>10N.222.47.A8 TTCTAGAGTCGGAAGAAGTTCACGAATGTTAGACACTTTCTTGTCGATAAGAAGAATGAACGGGCTATCTAGATCAACAGAACCGGCTTCTTGGTTGTTGATGAAGTAAGGAGATAGGTAACCGCGGTCGAATTGCATACCTTCAACTACGTCTAGCTCGTCTTGTAGAGCCTGACCTTCTTCAACCGTGATAACGCCATCACGACCTACTTTTTCCATCGCTTCAGCAATGATGTTACCTACTGTCGAATCAGAGTTCGCAGAGATAGTACCGACTTGCGCGATAGCTTTCGTGTCTGAACAAGGAACAGAAAGGTTCTTCAGCTCTTCAACAGCCGCGATAACCGCTTTGTCGATACCGCGCTTAAGATCCATTGGGTTCATGCCAGCAGCAACCGCTTTTAGGCCTTCAGCGATAATAGACTGAGCCAATACTGTTGCTGTCGTCGTCCC

>10N.222.47.A9 TTCTAGAGTCGGAAGAAGTTCACGAATGTTAGACACTTTCTTGTCGATAAGAAGAATGAACGGGCTATCTAGATCAACAGAACCGGCTTCTTGGTTGTTGATGAAGTAAGGAGATAGGTAACCGCGGTCGAATTGCATACCTTCAACTACGTCTAGCTCGTCTTGTAGAGCCTGACCTTCTTCAACCGTGATAACGCCATCACGACCTACTTTTTCCATCGCTTCAGCAATGATGTTACCTACTGTCGAATCAGAGTTCGCAGAGATAGTACCGACTTGCGCGATAGCTTTCGTGTCTGAACAAGGAACAGAAAGGTTCTTCAGCTCTTCAACAGCCGCGATAACCGCTTTGTCGATACCGCGCATAAGATCCATTGGGTTCATGCCAGCAGCCACCGCTTTTAGGCCTTCAGCGATAATAGACTGAGCCAATACTGTTGCTGTCGTCGTCCC

>10N.222.49.G1

TTCTAATGTTGGCAACAACTCACGAATGTTCAAGATTTTCTTATCAACCAGTAAAATAAATGGGCTTTCTAGTTCAACACTGCCCGCTTCTTGGTTGTTGATGAAGTATGGTGATAAGTAACCACGGTCAAATTGCATACCTTCGACAACATCAAGTTCGTCTTGTAGTGCTTGACCTTCTTCAACAGTGATTACACCATCACGACCTACTTTTTCCATTGCTTCAGCAATGATGTTACCTACACTCGTGTCAGAGTTGGCAGAAATTGTACCTACTTGAGCAATCGCTTTAGTGTCAGCACATGGTACAGACAGCTCTTTCAATGCTTCAACTGCTGCGATAACCGCTTTATCGATTCCGCGCTTAAGATCCATCGGATTCATACCCGCAGCAACTGCTTTTAGGCCTTCAGTGATGATCGCTTGCGCCAACACTGTTGCCGTCGTCGTCCC

>10N.222.49.F3 TTCTAGAGTCGGAAGAAGTTCACGGATGTTCGAAACTTTCTTGTCGATAAGAAGGATGAATGGGCTTTCTAGATCAACAGAACCTGCTTCTTGGTTGTTGATGAAGTAAGGAGATAGGTAACCGCGGTCGAACTGCATACCTTCAACTACGTCTAGCTCGTCTTGTAGAGCCTGACCTTCTTCAACCGTGATAACGCCATCACGACCTACTTTTTCCATCGCTTCAGCAATGATGTTACCTACTGTCGAATCAGAGTTCGCAGAGATAGTACCTACTTGCGCGATAGCTTTCGTGTCTGAACAAGGAACAGAAAGGTTCTTCAGCTCTTCAACAGCCGCGATAACCGCTTTGTCGATGCCGCGCTTAAGATCCATTGGGTTCATGCCAGCAGCAACCGCTTTTAGGCCTTCAGCGATAATAGACTGAGCCAATACTGTTGCTGTCGTCGTCCC

>10N.222.50.F10 TTCTAGAGTCGGAAGAAGTTCACGGATGTTCGACACTTTCTTGTCGATAAGAAGAATGAACGGGCTATCTAGATCAACAGAACCGGCTTCTTGGTTGTTGATGAAGTAAGGAGATAGGTAACCGCGGTCGAACTGCATACCTTCAACTACGTCTAACTCGTCTTGCAGAGCCTGACCTTCTTCAACCGTGATAACGCCATCACGACCTACTTTTTCCATCGCTTCAGCAATGATGTTACCTACTGTCGAATCAGAGTTCGCAGAGATAGTACCTACTTGCGCGATAGCTTTTGTGTCTGAACAAGGAACAGAAAGGTTCTTCAGCTCTTCAACAGCCGCGATAACCGCTTTGTCGATGCCGCGCTTAAGATCCATTGGGTTCATGCCAGCAGCAACCGCTTTTAGGCCTTCAGCGATAATAGACTGAGCCAATACTGTTGCTGTCGTCGTCCC

>10N.222.51.B5 TTCTAGAGTCGGAAGAAGTTCACGGATGTTCGACACTTTCTTGTCGATAAGAAGAATGAACGGGCTATCTAGATCAACAGAACCGGCTTCTTGGTTATTGATGAAGTAAGGAGATAGGTAACCGCGGTCGAACTGCATACCTTCAACTACGTCTAGCTCATCTTGTAGAGCCTGACCTTCTTCAACCGTGATAACGCCATCACGACCTACTTTTTCCATCGCTTCAGCAATGATGTTACCTACTGTCGAATCAGAGTTCGCAGAGATAGTACCTACTTGCGCGATAGCTTTCGTGTCTGAACAAGGAACAGAAAGGTTCTTCAGCTCTTCAACAGCCGCGATAACCGCTTTGTCGATGCCGCGCTTAAGATCCATTGGGTTCATGCCAGCAGCAACCGCTTTTAGGCCTTCAGCGATAATAGACTGAGCCAATACTGTTGCTGTCGTCGTCCC

>10N.222.51.D4 TTCTAGAGTCGGAAGCAGTTCACGGATGTTAGACACTTTCTTGTCGATAAGAAGAATGAATGGGCTTTCTAGATCAACAGAACCGGCTTCTTGGTTGTTGATGAAGTAAGGAGATAGGTATCCGCGGTCGAACTGCATACCTTCTACTACGTCTAGCTCATCTTGTAGAGCCTGACCTTCTTCAACCGTGATAACGCCATCACGACCTACTTTTTCCATCGCTTCAGCAATGATGTTACCTACTGTCGAATCAGAGTTCGCAGAGATAGTACCTACTTGCGCGATAGCTTTCGTGTCTGAACAAGGAACAGAAAGGTTCTTCAACTCTTCAACAGCCGCGATAACCGCTTTGTCGATGCCGCGCTTAAGATCCATTGGGTTCATGCCAGCAGCAACCGCTTTTAGGCCTTCTGCGATAATAGACTGAGCCAATACTGTAGCTGTCGTCGTCCC

>10N.222.52.A6 TTCTAGAGTCGGAAGAAGTTCACGGATGTTCGACACTTTCTTGTCGATAAGAAGAATGAACGGGCTATCTAGATCAACAGAACCGGCTTCTTGGTTATTGATGAAGTAAGGAGATAGGTAACCGCGGTCGAACTGCATACCTTCAACTACGTCTAGCTCATCTTGTAGAGCCTGACCTTCTTCAACCGTGATAACGCCATCACGACCTACTTTTTCCATCGCTTCAGCAATGATGTTACCTACTGTCGAATCAGAGTTCGCAGAGATAGTACCTACTTGCGCGATAGCTTTCGTGTCTGAACAAGGAACAGAAAGGTTCTTCAGCTCTTCAACAGCCGCGATAACCGCTTTGTCGATGCCGCGCTTAAGATCCATTGGGTTCATGCCAGCAGCAACCGCTTTTAGGCCTTCAGCGATAATAGACTGAGCCAATACTGTTGCTGTCGTCGTCCC

>10N.222.52.C6 TTCTAGAGTCGGAAGAAGTTCACGGATGTTCGACACTTTCTTGTCGATAAGAAGAATGAACGGGCTATCTAGATCAACAGAACCGGCTTCTTGGTTATTGATGAAGTAAGGAGATAGGTAACCGCGGTCGAACTGCATACCTTCAACTACGTCTAGCTCATCTTGTAGAGCCTGACCTTCTTCAACCGTGATAACGCCATCACGACCTACTTTTTCCATCGCTTCAGCAATGATGTTACCTACTGTCGAATCAGAGTTCGCAGAGATAGTACCTACTTGCGCGATAGCTTTCGTGTCTGAACAAGGAACAGAAAGGTTCTTCAGCTCTTCAACAGCCGCGATAACCGCTTTGTCGATGCCGCGCTTAAGATCCATTGGGTTCATGCCAGCAGCAACCGCTTTTAGGCCTTCAGCGATAATAGACTGAGCCAATACTGTTGCTGTCGTCGTCCC

>10N.222.52.E10 TTCTAGAGTCGGAAGAAGTTCACGGATGTTCGACACTTTCTTGTCGATAAGAAGAATGAACGGGCTATCTAGATCAACAGAACCGGCTTCTTGGTTGTTGATGAAGTAAGGAGATAGGTAACCGCGGTCGAACTGCATGCCTTCAACTACGTCTAGCTCGTCTTGTAGAGCCTGACCTTCTTCAACTGTGATAACGCCATCACGACCTACTTTTTCCATCGCTTCAGCAATGATGTTACCCACTGTCGAATCAGAGTTCGCAGAGATAGTACCTACTTGCGCGATCGCTTTCGTGTCTGAACAAGGAACAGAAAGGTTCTTCAGCTCTTCAACAGCCGCGATAACCGCTTTGTCGATGCCGCGCTTAAGATCCATTGGGTTCATGCCAGCAGCAACCGCTTTTAGGCCTTCAGCGATAATAGACTGAGCCAATACTGTTGCTGTCGTCGTCCC

>10N.222.53.H2 TTCTAGAGTCGGAAGAAGCTCACGAATGTTCGAAATTTTCTTGTCGATAAGAAGAATGAATGGGCTTTCTAGATCAACAGAGCCCGCTTCTTGGTTGTTGATGAAGTAAGGAGACAGGTAACCGCGATCGAACTGCATACCTTCAACTACGTCTAGCTCGTCTTGCAGAGCCTGACCTTCTTCAACCGTGATTACGCCATCACGGCCTACTTTTTCCATCGCTTCAGCAATGATGTTACCGACTGTCGAATCAGAGTTCGCAGAGATAGTACCGACTTGAGCAATCGCTTTACTGTCTGAACATGGCACAGAAAGGTTCTTCAGCTCTTCAACAGCCGCGATAACTGCTTTGTCGATACCGCGCTTAAGATCCATTGGGTTCATACCCGCAGCAACGGCTTTAGGCCTTCAGCGATAATCGACTGAGCCAATACTGTTGGGTGTCGTCGTCCC

>10N.222.54.C10 TTCTAGAGTCGGAAGAAGTTCACGGATGTTCGACACTTTCTTGTCGATAAGAAGAATGAACGGGCTATCTAGATCAACAGAACCGGCTTCTTGGTTGTTGATGAAGTAAGGAGATAGGTAACCGCGGTCGAACTGCATACCTTCAACTACGTCTAACTCGTCTTGCAGAGCCTGACCTTCTTCAACCGTGATAACGCCATCACGACCTACTTTTTCCATCGCTTCAGCAATGATGTTACCTACTGTCGAATCAGAGTTCGCAGAGATAGTACCTACTTGCGCGATAGCTTTTGTGTCTGAACAAGGAACAGAAAGGTTCTTCAGCTCTTCAACAGCCGCGATAACCGCTTTGTCGATGCCGCGCTTAAGATCCATTGGGTTCATGCCAGCAGCAACCGCTTTTAGGCCTTCAGCGATAATAGACTGAGCCAATACTGTTGCTGTCGTCGTCCC

>10N.222.54.F3 TTCTAGAGTCGGAAGAAGTTCACGGATGTTCGACACTTTCTTGTCGATAAGAAGAATGAACGGGCTATCTAGATCAACAGAACCGGCTTCTTGGTTGTTGATGAAGTAAGGAGATAGGTAACCGCGGTCGAACTGCATACCTTCAACTACGTCTAACTCGTCTTGCAGAGCCTGACCTTCTTCAACCGTGATAACGCCATCACGACCTACTTTTTCCATCGCTTCAGCAATGATGTTACCTACTGTCGAATCAGAGTTCGCAGAGATAGTACCTACTTGCGCGATAGCTTTTGTGTCTGAACAAGGAACAGAAAGGTTCTTCAGCTCTTCAACAGCCGCGATAACCGCTTTGTCGATGCCGCGCTTAAGATCCATTGGGTTCATGCCAGCAGCAACCGCTTTTAGGCCTTCAGCGATAATAGACTGAGCCAATACTGTTGCTGTCGTCGTCCC

>10N.222.56.C5 TTCTAGCGTTGGAAGAAGCTCACGAATGTTCGAGATTTTCTTATCAACTAGAAGGATGAATGGGCTTTCTAGATCAACAGAGCCTGCTTCTTGGTTGTTGATGAAGTAAGGAGATAGGTAACCGCGGTCGAACTGCATGCCTTCTACTACGTCTAGCTCATCTTGTAGAGCCTGACCTTCTTCAACGGTAATCACGCCATCACGGCCTACTTTTTCCATCGCTTCAGCAATGATGTTACCCACTGTTGCATCGGAGTTTGCAGAGATAGTACCTACCTGTGCGATAGCTTTGGTGTCGTTACATTCAACAGATAGTTCTTTTAGTTGCTCAACCGCTGCGATAACTGCTTTATCGATACCACGCTTAAGGTCCATTGGGTTCATACCAGCAGCAACCGCTTTTAACCCTTCGGTAATGATAGCTTGAGCAAGAACCGTTGCTGTCGTCGTCCC

>10N.261.45.A1 TTCTAGAGTCGGAAGAAGTTCACGGATATTTGAAACTTTCTTGTCGATTAGAAGGATGAATGGGCTTTCTAGATCAACAGAACCTGCTTCTTGGTTGTTGATGAAGTAAGGAGATAGGTAACCGCGGTCAAACTGCATACCTTCAACTACGTCTAGCTCGTCTTGTAGAGCCTGACCTTCTTCAACGGTGATAACACCATCACGGCCTACTTTTTCCATCGCTTCAGCAATGATGTTACCTACTGTCGCGTCAGAGTTTGCAGAGATAGTACCTACTTGTGCGATAGCTTTGGTATCTGCACATGGAACAGAAAGGCCTTTTAGCTCTTCAACTGCTGCAACAACGGCTTTGTCGATGCCGCGCTTAAGATCCATTGGGTTCATGCCAGCAGCAACGGCTTTTAGGCCTTCAGTGATGATAGCTTGAGCAAGAACAGTTGCCGTCGTCGTCCC

>10N.261.45.A6

TTCTAGAGTCGGAAGAAGTTCACGGATATTTGAAACTTTCTTGTCGATTAGAAGGATGAATGGGCTTTCTAGATCAACAGAACCTGCTTCTTGGTTGTTGATGAAGTAAGGAGATAGGTAACCGCGGTCAAACTGCATACCTTCAACTACGTCTAGCTCGTCTTGTAGAGCCTGACCTTCTTCAACGGTGATAACACCATCACGGCCTACTTTTTCCATCGCTTCAGCAATGATGTTACCTACTGTCGCGTCAGAGTTTGCAGAGATAGTACCTACTTGTGCGATAGCTTTGGTATCTGCACATGGAACAGAAAGGCCTTTTAGCTCTTCAACTGCTGCAACAACGGCTTTGTCGATGCCGCGCTTAAGATCCATTGGGTTCATGCCAGCAGCAACGGCTTTTAGGCCTTCAGTGATGATAGCTTGAGCAAGAACAGTTGCCGTCGTCGTCCC

>10N.261.45.A7 TTCTAGAGTCGGAAGAAGTTCACGGATATTTGAAACTTTCTTGTCGATTAGAAGGATGAATGGGCTTTCTAGATCAACAGAACCTGCTTCTTGGTTGTTGATGAAGTAAGGAGATAGGTAACCGCGGTCAAACTGCATACCTTCAACTACGTCTAGCTCGTCTTGTAGAGCCTGACCTTCTTCAACGGTGATAACACCATCACGGCCTACTTTTTCCATCGCTTCAGCAATGATGTTACCTACTGTCGCGTCAGAGTTTGCAGAGATAGTACCTACTTGTGCGATAGCTTTGGTATCTGCACATGGAACAGAAAGGCCTTTTAGCTCTTCAACTGCTGCAACAACGGCTTTGTCGATGCCGCGCTTAAGATCCATTGGGTTCATGCCAGCAGCAACGGCTTTTAGGCCTTCAGTGATGATAGCTTGAGCAAGAACAGTTGCCGTCGTCGTCCC

>10N.261.45.B6 TTCTAGAGTCGGAAGAAGTTCACGGATGTTCGACACTTTCTTGTCGATAAGAAGAATGAACGGGCTATCTAGATCAACAGAACCGGCTTCTTGGTTATTGATGAAGTAAGGAGATAGGTAACCGCGGTCGAACTGCATACCTTCAACTACGTCTAGCTCATCTTGTAGAGCCTGACCTTCTTCAACCGTGATAACGCCATCACGACCTACTTTTTCCATCGCTTCAGCAATGATGTTACCTACTGTCGAATCAGAGTTCGCAGAGATAGTACCTACTTGCGCGATAGCTTTCGTGTCTGAACAAGGAACAGAAAGGTTCTTCAGCTCTTCAACAGCCGCGATAACCGCTTTGTCGATGCCGCGCTAAAAATCCATTGGGTTCATGCCGCCCCCCCACGCTTTTAGCCTTCAGCGATAATAGACTGAGCCAATACTGTTGGCTGTCGTCGTCCC

>10N.261.45.C2 TTCTAGAGTCGGAAGAAGTTCACGGATATTTGAAACTTTCTTGTCGATTAGAAGGATGAATGGGCTTTCTAGATCAACAGAACCTGCTTCTTGGTTGTTGATGAAGTAAGGAGATAGGTAACCGCGGTCAAACTGCATACCTTCAACTACGTCTAGCTCGTCTTGTAGAGCCTGACCTTCTTCAACGGTGATAACACCATCACGGCCTACTTTTTCCATCGCTTCAGCAATGATGTTACCTACTGTCGCGTCAGAGTTTGCAGAGATAGTACCTACTTGTGCGATAGCTTTGGTATCTGCACATGGAACAGAAAGGCCTTTTAGCTCTTCAACTGCTGCAACAACGGCTTTGTCGATGCCGCGCTTAAGATCCATTGGGTTCATGCCAGCAGCAACGGCTTTTAGGCCTTCAGTGATGATAGCTTGAGCAAGAACAGTTGCCGTCGTCGTCCC

>10N.261.45.D2 TTCTAGAGTCGGAAGAAGTTCACGGATATTTGAAACTTTCTTGTCGATTAGAAGGATGAATGGGCTTTCTAGATCAACAGAACCTGCTTCTTGGTTGTTGATGAAGTAAGGAGATAGGTAACCGCGGTCAAACTGCATACCTTCAACTACGTCTAGCTCGTCTTGTAGAGCCTGACCTTCTTCAACGGTGATAACACCATCACGGCCTACTTTTTCCATCGCTTCAGCAATGATGTTACCTACTGTCGCGTCAGAGTTTGCAGAGATAGTACCTACTTGTGCGATAGCTTTGGTATCTGCACATGGAACAGAAAGGCCTTTTAGCTCTTCAACTGCTGCAACAACGGCTTTGTCGATGCCGCGCTTAAGATCCATTGGGTTCATGCCAGCAGCAACGGCTTTTAGGCCTTCAGTGATGATAGCTTGAGCAAGAACAGTTGCCGTCGTCGTCCC

>10N.261.45.E1 TTCTAGAGTCGGAAGAAGTTCACGGATATTTGAAACTTTCTTGTCGATTAGAAGGATGAATGGGCTTTCTAGATCAACAGAACCTGCTTCTTGGTTGTTGATGAAGTAAGGAGATAGGTAACCGCGGTCAAACTGCATACCTTCAACTACGTCTAGCTCGTCTTGTAGAGCCTGACCTTCTTCAACGGTGATAACACCATCACGGCCTACTTTTTCCATCGCTTCAGCAATGATGTTACCTACTGTCGCGTCAGAGTTTGCAGAGATAGTACCTACTTGTGCGATAGCTTTGGTATCTGCACATGGAACAGAAAGGCCTTTTAGCTCTTCAACTGCTGCAACAACGGCTTTGTCGATGCCGCGCTTAAGATCCATTGGGTTCATGCCAGCAGCAACGGCTTTTAGGCCTTCAGTGATGATAGCTTGAGCAAGAACAGTTGCCGTCGTCGTCCC

>10N.261.45.E2 TTCTAGAGTCGGAAGAAGTTCACGGATATTTGAAACTTTCTTGTCGATTAGAAGGATGAATGGGCTTTCTAGATCAACAGAACCTGCTTCTTGGTTGTTGATGAAGTAAGGAGATAGGTAACCGCGGTCAAACTGCATACCTTCAACTACGTCTAGCTCGTCTTGTAGAGCCTGACCTTCTTCAACGGTGATAACACCATCACGGCCTACTTTTTCCATCGCTTCAGCAATGATGTTACCTACTGTCGCGTCAGAGTTTGCAGAGATAGTACCTACTTGTGCGATAGCTTTGGTATCTGCACATGGAACAGAAAGGCCTTTTAGCTCTTCAACTGCTGCAACAACGGCTTTGTCGATGCCGCGCTTAAGATCCATTGGGTTCATGCCAGCAGCAACGGCTTTTAGGCCTTCAGTGATGATAGCTTGAGCAAGAACAGTTGCCGTCGTCGTCCC

>10N.261.45.G3 TTCTAGAGTCGGAAGAAGTTCACGGATATTTGAAACTTTCTTGTCGATTAGAAGGATGAATGGGCTTTCTAGATCAACAGAACCTGCTTCTTGGTTGTTGATGAAGTAAGGAGATAGGTAACCGCGGTCAAACTGCATACCTTCAACTACGTCTAGCTCGTCTTGTAGAGCCTGACCTTCTTCAACGGTGATAACACCATCACGGCCTACTTTTTCCATCGCTTCAGCAATGATGTTACCTACTGTCGCGTCAGAGTTTGCAGAGATAGTACCTACTTGTGCGATAGCTTTGGTATCTGCACATGGAACAGAAAGGCCTTTTAGCTCTTCAACTGCTGCAACAACGGCTTTGTCGATGCCGCGCTTAAGATCCATTGGGTTCATGCCAGCAGCAACGGCTTTTAGGCCTTCAGTGATGATAGCTTGAGCAAGAACAGTTGCCGTCGTCGTCCC

>10N.261.45.H4 TTCTAGAGTCGGAAGAAGTTCACGGATATTTGAAACTTTCTTGTCGATTAGAAGGATGAATGGGCTTTCTAGATCAACAGAACCTGCTTCTTGGTTGTTGATGAAGTAAGGAGATAGGTAACCGCGGTCAAACTGCATACCTTCAACTACGTCTAGCTCGTCTTGTAGAGCCTGACCTTCTTCAACGGTGATAACACCATCACGGCCTACTTTTTCCATCGCTTCAGCAATGATGTTACCTACTGTCGCGTCAGAGTTTGCAGAGATAGTACCTACTTGTGCGATAGCTTTGGTATCTGCACATGGAACAGAAAGGCCTTTTAGCTCTTCAACTGCTGCAACAACGGCTTTGTCGATGCCGCGCTTAAGATCCATTGGGTTCATGCCAGCAGCAACGGCTTTTAGGCCTTCAGTGATGATAGCTTGAGCAAGAACAGTTGCCGTCGTCGTCCC

>10N.261.46.A10

TTCTAGAGTCGGAAGAAGTTCACGGATGTTCGACACTTTCTTGTCGATAAGAAGGATAAACGGGCTTTCTAGATCAACAGAACCCGCTTCTTGGTTGTTGATGAAGTAAGGAGATAGGTAACCGCGGTCGAACTGCATACCTTCTACTACGTCTAGCTCGTCTTGCAGAGCCTGACCTTCTTCAACGGTGATAACGCCATCACGACCTACTTTTTCCATCGCTTCAGCAATGATGTTACCGACTGTCGCATCAGAATTTGCAGAGATAGTACCGACTTGCGCGATAGCTTTCGTGTCTGCACATGGCACAGAAAGGCCTTTTAACTCTTCAACCGCTGCGATAACCGCTTTGTCGATGCCGCGCTTAAGATCCATTGGGTTCATGCCAGCTGCTACCGCTTTCAGGCCTTCAGTGATAATGGCTTGAGCAAGAACTGTTGCTGTCGTCGTCCC

>10N.261.46.B8 TTCTAGAGTCGGAAGAAGTTCACGGATGTTCGACACTTTCTTATCGATAAGAAGAATGAACGGGCTATCTAGATCAACAGAACCGGCTTCTTGGTTGTTGATGAAGTAAGGAGATAGGTAACCGCGGTCGAACTGCATACCTTCAACTACGTCTAGCTCGTCTTGCAGAGCCTGACCTTCTTCAACCGTGATTACACCATCACGACCTACTTTTTCCATCGCTTCAGCAATGATGTTACCTACTGTCGAATCAGAGTTCGCAGAGATAGTACCTACTTGCGCGATAGCTTTCGTGTCTGAACAAGGAACAGAAAGGTTCTTCAGCTCTTCAACAGCCGCGATAACCGCTTTGTCGATGCCGCGCTTAAGATCCATTGGGTTCATGCCAGCAGCAACCGCTTTTAGGCCTTCAGCGATAATAGACTGAGCCAATACTGTTGCTGTCGTCGTCCC

>10N.261.46.C2

TTCCAGAGTCGGTAGCAATTCACGGATGTTTGATACTTTCTTGTCAACCAACAAGATGAATGGGCTATCTAAATCAATAGAACCAGACTCTTGGTTGTTAATGAAGTAAGGAGACAGGTAGCCGCGATCAAACTGCATACCTTCTACTACGTCTAGCTCGTCTTGAAGAGATTGGCCTTCTTCAACAGTGATAACACCGTCACGACCAACTTTTTCCATTGCTTCTGCAATCAGTTTACCCACTGTCTCATCAGAGTTTGCTGAGATAGTACCAACTTGCGCAATCGCTTTGGTATCTGCACATGGAACAGACAGTGCTTTCAACTCTTCAACAGCAGCGATGACTGCTTTGTCGATACCACGCTTCAGATCCATTGGGTTCATACCCGCTGCAACTGCTTTAAGACCTTCAGTAATGATAGATTGCGCGATAACCGTGGCAGTCGTCGTCCC

>10N.261.46.C8 TTCTAGAGTCGGAAGAAGTTCACGGATGTTCGACACTTTCTTGTCGATAAGAAGAATGAACGGGCTATCTAGATCAACAGAACCGGCTTCTTGGTTGTTGATGAAGTAAGGAGATAGGTAACCGCGGTCGAACTGCATACCTTCAACTACGTCTAGCTCATCTTGTAGAGCCTGACCTTCTTCAACCGTGATAACGCCATCACGACCTACTTTTTCCATCGCTTCAGCAATGATGTTACCCACTGTCGAATCAGAGTTCGCAGAGATAGTACCTACTTGCGCGATAGCTTTCGTGTCTGAACAAGGAACAGAAAGGTTCTTCAGCTCTTCAACAGCCGCGATAACCGCTTTGTCGATGCCGCGCTTAAGATCCATTGGGTTCATGCCAGCAGCAACCGCTTTTAGGCCTTCAGCGATAATAGACTGAGCCAATACTGTTGCTGTCGTCGTCCC

>10N.261.46.C10 TTCTAGAGTCGGAAGAAGTTCACGGATATTTGAAACTTTCTTGTCGATTAGAAGGATGAATGGGCTTTCTAGATCAACAGAACCTGCTTCTTGGTTGTTGATGAAGTAAGGAGATAGGTAACCGCGGTCAAACTGCATACCTTCAACTACGTCTAGCTCGTCTTGTAGAGCCTGACCTTCTTCAACGGTGATAACACCATCACGGCCTACTTTTTCCATCGCTTCAGCAATGATGTTACCTACTGTCGCGTCAGAGTTTGCAGAGATAGTACCTACTTGTGCGATAGCTTTGGTATCTGCACATGGAACAGAAAGGCCTTTTAGCTCTTCAACTGCTGCAACAACGGCTTTGTCGATGCCGCGCTTAAGATCCATTGGGTTCATGCCAGCAGCAACGGCTTTTAGGCCTTCAGTGATGATAGCTTGAGCAAGAACAGTTGCCGTCGTCGTCCC

>10N.261.46.D8 TTCTAGAGCAGGAAGAAGTTCACGGATGTTCGAAACTTTCTTGTCGATAAGAAGGATGAATGGGCTTTCTAGATCAACAGAACCTGCTTCTTGGTTGTTGATGAAGTAAGGAGATAGGTAACCGCGGTCGAACTGCATACCTTCAACTACGTCTAGCTCGTCTTGCAGAGCCTGACCTTCTTCAACCGTGATTACGCCATCACGACCTACTTTTTCCATCGCTTCAGCAATGATGTTACCTACTGTCGAATCAGAGTTCGCAGAGATAGTACCTACTTGCGCGATAGCTTTCGTGTCTGAACAAGGAACAGAAAGGTTCTTCAGCTCTTCAACAGCCGCGATAACCGCTTTGTCGATGCCGCGCTTAAGATCCATTGGGTTCATGCCAGCAGCAACCGCTTTTAGGCCTTCCGCGATAATAGACTGAGCCAATACTGTTGCGGTCGTCGTCCC

>10N.261.46.D10 TTCTAGAGTCGGAAGAAGTTCACGGATATTTGAAACTTTCTTGTCGATTAGAAGGATGAATGGGCTTTCTAGATCAACAGAACCTGCTTCTTGGTTGTTGATGAAGTAAGGAGATAGGTAACCGCGGTCAAACTGCATACCTTCAACTACGTCTAGCTCGTCTTGTAGAGCCTGACCTTCTTCAACGGTGATAACACCATCACGGCCTACTTTTTCCATCGCTTCAGCAATGATGTTACCTACTGTCGCGTCAGAGTTTGCAGAGATAGTACCTACTTGTGCGATAGCTTTGGTATCTGCACATGGAACAGAAAGGCCTTTTAGCTCTTCAACTGCTGCAACAACGGCTTTGTCGATGCCGCGCTTAAGATCCATTGGGTTCATGCCAGCAGCAACGGCTTTTAGGCCTTCAGTGATGATAGCTTGAGCAAGAACAGTTGCCGTCGTCGTCCC

>10N.261.46.D11 TTCTAGAGTCGGAAGAAGTTCACGGATGTTCGACACTTTCTTATCGATAAGAAGAATGAACGGGCTATCTAGATCAACAGAACCGGCTTCTTGGTTGTTGATGAAGTAAGGAGATAGGTAACCGCGGTCGAACTGCATACCTTCAACTACGTCTAGCTCGTCTTGCAGAGCCTGACCTTCTTCAACCGTGATTACAGCATCACGACCTACTTTTTCCATCGCTTCAGCAATGATGTTACCTACTGTCGAATCAGAGTTCGCAGAGATAGTACCTACTTGCGCGATAGCTTTCGTGTCTGAACAAGGAACAGAAAGGTTCTTCAGCTCTTCAACAGCCGCGATAACCGCTTTGTCGATGCCGCGCTTAAGATCCATTGGGTTCATGCCAGCAGCAACCGCTTTTAGGCCTTCAGCGATAATAGACTGAGCCAATACTGTTGCTGTCGTCGTCCC

>10N.261.46.E8 TTCTAGAGTCGGAAGAAGTTCACGGATATTTGAAACTTTCTTGTCGATTAGAAGGATGAATGGGCTTTCTAGATCAACAGAACCTGCTTCTTGGTTGTTGATGAAGTAAGGAGATAGGTAACCGCGGTCAAACTGCATACCTTCAACTACGTCTAGCTCGTCTTGTAGAGCCTGACCTTCTTCAACGGTGATAACACCATCACGGCCTACTTTTTCCATCGCTTCAGCAATGATGTTACCTACTGTCGCGTCAGAGTTTGCAGAGATAGTACCTACTTGTGCGATAGCTTTGGTATCTGCACATGGAACAGAAAGGCCTTTTAGCTCTTCAACTGCTGCAACAACGGCTTTGTCGATGCCGCGCTTAAGATCCATTGGGTTCATGCCAGCAGCAACGGCTTTTAGGCCTTCAGTGATGATAGCTTGAGCAAGAACAGTTGCCGTCGTCGTCCC

>10N.261.46.E12 TTCTAGAGTCGGAAGAAGTTCACGGATATTTGAAACTTTCTTGTCGATTAGAAGGATGAATGGGCTTTCTAGATCAACAGAACCTGCTTCTTGGTTGTTGATGAAGTAAGGAGATAGGTAACCGCGGTCAAACTGCATACCTTCAACTACGTCTAGCTCGTCTTGTAGAGCCTGACCTTCTTCAACGGTGATAACACCATCACGGCCTACTTTTTCCATCGCTTCAGCAATGATGTTACCTACTGTCGCGTCAGAGTTTGCAGAGATAGTACCTACTTGTGCGATAGCTTTGGTATCTGCACATGGAACAGAAAGGCCTTTTAGCTCTTCAACTGCTGCGACAACCGCTTTGTCGATGCCGCGCTTAAGATCCATTGGGTTCATGCCAGCAGCAACGTGCTTTAGCCTTCAGTGATGATAGCTTGGAGCAAGAACAGTTGCCGTCGTCGTCCC

>10N.261.46.F6 TTCTAGAGTCGGAAGAAGTTCACGGATGTTCGACACTTTCTTGTCGATAAGAAGAATGAACGGGCTATCTAGATCAACAGAACCGGCTTCTTGGTTATTGATGAAGTAAGGAGATAGGTAACCGCGGTCGAACTGCATACCTTCAACTACGTCTAGCTCATCTTGTAGAGCCTGACCTTCTTCAACCGTGATAACGCCATCACGACCTACTTTTTCCATCGCTTCAGCAATGATGTTACCTACTGTCGAATCAGAGTTCGCAGAGATAGTACCTACTTGCGCGATAGCTTTCGTGTCTGAACAAGGAACAGAAAGGTTCTTCAGCTCTTCAACAGCCGCGATAACCGCTTTGTCGATGCCGCGCTTAAGATCCATTGGGTTCATGCCAGCAGCAACGGCTTTTAGGCCTTCAGCGATAATAGACTGAGCCAATACTGTTGCTGTCGTCGTCCC

>10N.261.46.F12 TTCTAGAGTCGGAAGAAGTTCACGGATATTTGAAACTTTCTTGTCGATTAGAAGGATGAATGGGCTTTCTAGATCAACAGAACCTGCTTCTTGGTTGTTGATGAAGTAAGGAGATAGGTAACCGCGGTCAAACTGCATACCTTCAACTACGTCTAGCTCGTCTTGTAGAGCCTGACCTTCTTCAACGGTGATAACACCATCACGGCCTACTTTTTCCATCGCTTCAGCAATGATGTTACCTACTGTCGCGTCAGAGTTTGCAGAGATAGTACCTACTTGTGCGATAGCTTTGGTATCTGCACATGGAACAGAAAGGCCTTTTAGCTCTTCAACTGCTGCAACAACGGCTTTGTCGATGCCGCGCTTAAGATCCATTGGGTTCATGCCAGCAGCAACGGCTTTTAGGCCTTCAGTGATGATAGCTTGAGCAAGAACAGTTGCCGTCGTCGTCCC

>10N.261.46.G12 TTCTAGTGCTGGAAGTAGCTCACGAATGTTTGATACTTTCTTATCGATGAGAAGAATGAATGGGCTTTCTAGGTCTACACTACCCGCTTCTTGGTTGTTGATGAAGTAAGGAGATAGGTAACCGCGATCGAACTGCATACCTTCAACTACGTCTAGCTCGTCTTGAAGAGCCTGACCTTCTTCAACAGTGATAACACCTTCACGGCCTACTTTTTCCATCGCTTCAGCCATGATGTTACCCAGAGTCACATCAGAGTTTGCAGAGATAGTACCTACCTCAGCGATAGCTTTAGTATCAGCACAGAGTTGAGATAGGTTCTTAAGCTCTTCAACTGCCGCCGATCCCTGTCTGTCGATACCACGCTAAAAATCCATTGGGTTCATACCCGCAGCCACGCCGTAAACACCATTAGTGAAGATAGATAGAGCACGTATGTTGGGGTTCGTCGCCCC

>10N.261.46.H1

TTCTAGAGTCGGAAGAAGTTCACGGATATTTGAAACTTTCTTGTCGATTAGAAGGATGAATGGGCTTTCTAGATCAACAGAACCTGCTTCTTGGTTGTTGATGAAGTAAGGAGATAGGTAACCGCGGTCAAACTGCATACCTTCAACTACGTCTAGCTCGTCTTGTAGAGCCTGACCTTCTTCAACGGTGATAACACCATCACGGCCTACTTTTTCCATCGCTTCAGCAATGATGTTACCTACTGTCGCGTCAGAGTTTGCAGAGATAGTACCTACTTGTGCGATAGCTTTGGTATCTGCACATGGAACAGAAAGGCCTTTTAGCTCTTCAACTGCTGCAACAACGGCTTTGTCGATGCCGCGCTTAAGATCCATTGGGTTCATGCCAGCAGCAACGGCTTTTAGGCCTTCAGTGATGATAGCTTGAGCAAGAACAGTTGCCGTCGTCGTCCC

>10N.261.47.B2 TTCTAGAGTCGGAAGAAGCTCACGAATGTTCGAAATTTTCTTGTCGATAAGAAGAATGAATGGGCTTTCTAGATCAACAGAGCCCGCTTCTTGGTTGTTGATGAAGTAAGGAGACAGGTAACCGCGATCGAACTGCATACCTTCAACTACGTCTAGCTCGTCTTGCAGAGCCTGACCTTCTTCAACCGTGATTACGCCATCACGGCCTACTTTTTCCATCGCTTCAGCAATGATGTTACCGACTGTCGAATCAGAGTTCGCAGAGATAGTACCGACTTGAGCAATCGCTTTACTGTCTGAACATGGCACAGAAAGGTTCTTCAGCTCTTCAACAGCCGCGATAACTGCTTTGTCGATACCGCGCTTAAGATCCATTGGGTTCATACCCGCAGCAACGGCTTTTAGGCCTTCAGCGATAATCGACTGAGCCAATACGGTTGCTGTCGTCGTCCC

>10N.261.47.C4

TTCTAGAGTCGGAAGAAGTTCACGGATATTTGAAACTTTCTTGTCGATTAGAAGGATGAATGGGCTTTCTAGATCAACAGAACCTGCTTCTTGGTTGTTGATGAAGTAAGGAGATAGGTAACCGCGGTCAAACTGCATACCTTCAACTACGTCTAGCTCGTCTTGTAGAGCCTGACCTTCTTCAACGGTGATAACACCATCACGGCCTACTTTTTCCATCGCTTCAGCAATGATGTTACCTACTGTCGCGTCAGAGTTTGCAGAGATAGTACCTACTTGTGCGATAGCTTTGGTATCTGCACATGGAACAGAAAGGCCTTTTAGCTCTTCAACTGCTGCAACAACGGCTTTGTCGATGCCGCGCTTAAGATCCATTGGGTTCATGCCAGCAGCAACGGCTTTTAGGCCTTCAGTGATGATAGCTTGAGCAAGAACAGTTGCCGTCGTCGTCCC

>10N.261.47.C5 TTCTAGAGTCGGAAGAAGTTCACGGATATTTGAAACTTTCTTGTCGATTAGAAGGATGAATGGGCTTTCTAGATCAACAGAACCTGCTTCTTGGTTGTTGATGAAGTAAGGAGATAGGTAACCGCGGTCAAACTGCATACCTTCAACTACGTCTAGCTCGTCTTGTAGAGCCTGACCTTCTTCAACGGTGATAACACCATCACGGCCTACTTTTTCCATCGCTTCAGCAATGATGTTACCTACTGTCGCGTCAGAGTTTGCAGAGATAGTACCTACTTGTGCGATAGCTTTGGTATCTGCACATGGAACAGAAAGGCCTTTTAGCTCTTCAACTGCTGCAACAACGGCTTTGTCGATGCCGCGCTTAAGATCCATTGGGTTCATGCCAGCAGCAACGGCTTTTAGGCCTTCAGTGATGATAGCTTGAGCAAGAACAGTTGCCGTCGTCGTCCC

>10N.261.47.E5 TTCTAGAGTCGGAAGAAGTTCACGGATATTTGAAACTTTCTTGTCGATTAGAAGGATGAATGGGCTTTCTAGATCAACAGAACCTGCTTCTTGGTTGTTGATGAAGTAAGGAGATAGGTAACCGCGGTCAAACTGCATACCTTCAACTACGTCTAGCTCGTCTTGTAGAGCCTGACCTTCTTCAACGGTGATAACACCATCACGGCCTACTTTTTCCATCGCTTCAGCAATGATGTTACCTACTGTCGCGTCAGAGTTTGCAGAGATAGTACCTACTTGTGCGATAGCTTTGGTATCTGCACATGGAACAGAAAGGCCTTTTAGCTCTTCAACTGCTGCAACAACGGCTTTGTCGATGCCGCGCTTAAGATCCATTGGGTTCATGCCAGCCGCCACGGCTTTTAGGCCTTCAGTGATGATAGCTTGAGCAAGAACAGTTGCCGTCGTCGTCCC

>10N.261.47.E10 TTCTAGAGTCGGAAGAAGTTCACGGATATTTGAAACTTTCTTGTCGATTAGAAGGATGAATGGGCTTTCTAGATCAACAGAACCTGCTTCTTGGTTGTTGATGAAGTAAGGAGATAGGTAACCGCGGTCAAACTGCATACCTTCAACTACGTCTAGCTCGTCTTGTAGAGCCTGACCTTCTTCAACGGTGATAACACCATCACGGCCTACTTTTTCCATCGCTTCAGCAATGATGTTACCTACTGTCGCGTCAGAGTTTGCAGAGATAGTACCTACTTGTGCGATAGCTTTGGTATCTGCACATGGAACAGAAAGGCCTTTTAGCTCTTCAACTGCTGCAACAACGGCTTTGTCGATGCCGCGCTTAAGATCCATTGGGTTCATGCCAGCAGCAACGGCTTTTAGGCCTTCAGTGATGATAGCTTGAGCAAGAACAGTTGCCGTCGTCGTCCC

>10N.261.47.F7

TTCTAGAGTCGGAAGAAGTTCACGGATATTTGAAACTTTCTTGTCGATTAGAAGGATGAATGGGCTTTCTAGATCAACAGAACCTGCTTCTTGGTTGTTGATGAAGTAAGGAGATAGGTAACCGCGGTCAAACTGCATACCTTCAACTACGTCTAGCTCGTCTTGTAGAGCCTGACCTTCTTCAACGGTGATAACACCATCACGGCCTACTTTTTCCATCGCTTCAGCAATGATGTTACCTACTGTCGCGTCAGAGTTTGCAGAGATAGTACCTACTTGTGCGATAGCTTTGGTATCTGCACATGGAACAGAAAGGCCTTTTAGCTCTTCAACTGCTGCAACAACGGCTTTGTCGATGCCGCGCTTAAGATCCATTGGGTTCATGCCAGCAGCAACGGCTTTTAGGCCTTCAGTGATGATAGCTTGAGCAAGAACAGTTGCCGTCGTCGTCCC

>10N.261.48.D6 TTCTAGAGTCGGAAGCAGTTCACGGATGTTCGATACTTTCTTGTCGATAAGAAGAATGAATGGGCTTTCTAGATCAACAGAACCCGCTTCTTGGTTGTTGATGAAGTAAGGAGATAGGTAACCGCGGTCGAACTGCATACCTTCCACTACGTCTAGCTCGTCTTGTAGAGCCTGACCTTCTTCAACTGTGATAACGCCATCACGACCTACTTTTTCCATCGCTTCAGCAATGATGTTACCTACTGTCGAATCAGAGTTCGCAGAGATAGTACCTACTTGAGCGATAGCTTTCGTGTCTGAACAAGGAACAGAAAGGTTCTTCAGCTCTTCAACAGCCGCGATAACCGCTTTGTCGATGCCGCGCTTAAGATCCATTGGGTTCATGCCAGCAGCAACCGCTTTTAGGCCTTCAGCAATAATAGACTGAGCCAATACTGTTGCTGTCGTCGTCCC

>10N.261.48.E7 TTCTAGAGTCGGAAGAAGCTCACGAATGTTCGAAATTTTCTTGTCGATAAGAAGAATGAATGGGCTTTCTAGATCAACAGAGCCCGCTTCTTGGTTGTTGATGAAGTAAGGAGACAGGTAACCGCGATCGAACTGCATACCTTCAACTACGTCTAGCTCGTCTTGCAGAGCCTGACCTTCTTCAACCGTGATTACGCCATCACGGCCTACTTTTTCCATCGCTTCAGCAATGATGTTACCGACTGTCGAATCAGAGTTCGCAGAGATAGTACCGACTTGAGCAATCGCTTTACTGTCTGAACATGGCACAGAAAGGTTCTTCAGCTCTTCAACAGCCGCGATAACTGCTTTGTCGATACCGCGCTTAAGATCCATTGGGTTCATACCCGCAGCAACGGCTTTTAGGCCTTCAGCGATAATCGACTGAGCCAATACGGTTGCTGTCGTCGTCCC

>10N.261.48.F7 TTCTAGAGTCGGAAGAAGTTCACGGATGTTCGACACTTTCTTGTCGATAAGAAGAATGAACGGGCTATCTAGATCAACAGAACCGGCTTCTTGGTTGTTGATGAAGTAAGGAGATAGGTAACCGCGGTCGAACTGCATACCTTCAACTACGTCTAGCTCGTCTTGCAGAGCCTGACCTTCTTCAACCGTGATTACACCATCACGACCTACTTTTTCCATCGCTTCAGCAATGATGTTACCTACTGTCGAATCAGAGTTCGCAGAGATAGTACCTACTTGCGCGATAGCTTTCGTGTCTGAACAAGGAACAGATAGGTTCTTCAGCTCTTCAACAGCTGCGATAACCGCTTTGTCGATGCCGCGCTTAAGATCCATTGGGTTCATGCCAGCAGCAACCGCTTTTAGGCCTTCAGCGATAATAGACTGAGCCAATACTGTCGCTGTCGTCGTCCC

>10N.261.48.H9 TTCTAGAGTCGGAAGAAGTTCACGGATGTTCGACACTTTCTTGTCGATAAGAAGAATGAACGGGCTATCTAGATCAACAGAACCGGCTTCTTGGTTGTTGATGAAGTAAGGAGATAGGTAACCGCGGTCGAACTGCATACCTTCAACTACGTCTAACTCGTCTTGCAGAGCCTGACCTTCTTCAACCGTGATAACGCCATCACGACCTACTTTTTCCATCGCTTCAGCAATGATGTTACCTACTGTCGAATCAGAGTTCGCAGAGATAGTACCTACTTGCGCGATAGCTTTTGTGTCTGAACAAGGAACAGAAAGGTTCTTCAGCTCTTCAACAGCCGCGATAACCGCTTTGTCGATGCCGCGCTTAAGATCCATTGGGTTCATGCCAGCAGCAACCGCTTTTAGGCCTTCAGCGATAATAGACTGAGCCAATACTGTTGCTGTCGTCGTCCC

>10N.261.49.A3

TTCTAGAGTCGGAAGAAGTTCACGGATATTTGAAACTTTCTTGTCGATTAGAAGGATGAATGGGCTTTCTAGATCAACAGAACCTGCTTCTTGGTTGTTGATGAAGTAAGGAGATAGGTAACCGCGGTCAAACTGCATACCTTCAACTACGTCTAGCTCGTCTTGTAGAGCCTGACCTTCTTCAACGGTGATAACACCATCACGGCCTACTTTTTCCATCGCTTCAGCAATGATGTTACCTACTGTCGCGTCAGAGTTTGCAGAGATAGTACCTACTTGTGCGATAGCTTTGGTATCTGCACATGGAACAGAAAGGCCTTTTAGCTCTTCAACTGCTGCAACAACGGCTTTGTCGATGCCGCGCTTAAGATCCATTGGGTTCATGCCAGCAGCAACGGCTTTTAGGCCTTCAGTGATGATAGCTTGAGCAAGAACAGTTGCCGTCGTCGTCCC

>10N.261.49.C9 TTCTAGTGTTGGAAGTAGCTCACGAATGTTTGATACTTTCTTATCGATAAGAAGAATGAATGGGCTTTCTAGGTCTACGCTACCCGCTTCTTGGTTGTTGATGAAGTAAGGAGATAGGTAACCGCGATCGAACTGCATACCTTCAACTACGTCTAGCTCGTCTTGAAGAGCCTGACCTTCTTCAACAGTGATAACACCATCACGGCCTACTTTTTCCATCGCTTCAGCAATGATGTTACCTACAGTCACATCAGAGTTTGCAGAGATAGTACCTACCTGAGCGATAGCTTTAGTATCAGCACAAGGTTGAGATAGGTTCTTAAGCTCTTCAACTGCAGCAATCACTGCTTTGTCGATACCACGCTTAAGATCCATTGGGTTCATACCAGCAGCGACAGCCTTAAGGCCTTCAGTGATGATAGATTGAGCCAGTACTGTTGCAGTCGTCGTCCC

>10N.261.49.C12 TTCTAGAGTCGGAAGAAGTTCACGGATATTTGAAACTTTCTTGTCGATTAGAAGGATGAATGGGCTTTCTAGATCAACAGAACCTGCTTCTTGGTTGTTGATGAAGTAAGGAGATAGGTAACCGCGGTCAAACTGCATACCTTCAACTACGTCTAGCTCGTCTTGTAGAGCCTGACCTTCTTCAACGGTGATAACACCATCACGGCCTACTTTTTCCATCGCTTCAGCAATGATGTTACCTACTGTCGCGTCAGAGTTTGCAGAGATAGTACCTACTTGTGCGATAGCTTTGGTATCTGCACATGGAACAGAAAGGCCTTTTAGCTCTTCAACTGCTGCAACAACGGCTTTGTCGATGCCGCGCTTAAGATCCATTGGGTTCATGCCAGCAGCAACGGCTTTTAGGCCTTCAGTGATGATAGCTTGAGCAAGAACAGTTGCCGTCGTCGTCCC

>10N.261.49.D3 TTCTAGAGTCGGAAGAAGTTCACGGATATTTGAAACTTTCTTGTCGATTAGAAGGATGAATGGGCTTTCTAGATCAACAGAACCTGCTTCTTGGTTGTTGATGAAGTAAGGAGATAGGTAACCGCGGTCAAACTGCATACCTTCAACTACGTCTAGCTCGTCTTGTAGAGCCTGACCTTCTTCAACGGTGATAACACCATCACGGCCTACTTTTTCCATCGCTTCAGCAATGATGTTACCTACTGTCGCGTCAGAGTTTGCAGAGATAGTACCTACTTGTGCGATAGCTTTGGTATCTGCACATGGAACAGAAAGGCCTTTTAGCTCTTCAACTGCTGCAACAACGGCTTTGTCGATGCCGCGCTTAAGATCCATTGGGTTCATGCCAGCAGCAACGGCTTTTAGGCCTTCAGTGATGATAGCTTGAGCAAGAACAGTTGCCGTCGTCGTCCC

>10N.261.49.D12 TTCTAGAGTCGGAAGAAGTTCACGGATATTTGAAACTTTCTTGTCGATTAGAAGGATGAATGGGCTTTCTAGATCAACAGAACCTGCTTCTTGGTTGTTGATGAAGTAAGGAGATAGGTAACCGCGGTCAAACTGCATACCTTCAACTACGTCTAGCTCGTCTTGTAGAGCCTGACCTTCTTCAACGGTGATAACACCATCACGGCCTACTTTTTCCATCGCTTCAGCAATGATGTTACCTACTGTCGCGTCAGAGTTTGCAGAGATAGTACCTACTTGTGCGATAGCTTTGGTATCTGCACATGGAACAGAAAGGCCTTTTAGCTCTTCAACTGCTGCAACAACGGCTTTGTCGATGCCGCGCTTAAGATCCATTGGGTTCATGCCAGCAGCAACGGCTTTTAGGCCTTCAGTGATGATAGCTTGAGCAAGAACAGTTGCCGTCGTCGTCCC

>10N.261.49.E11 TTCTAGAGTCGGAAGAAGTTCACGGATATTTGAAACTTTCTTGTCGATTAGAAGGATGAATGGGCTTTCTAGATCAACAGAACCTGCTTCTTGGTTGTTGATGAAGTAAGGAGATAGGTAACCGCGGTCAAACTGCATACCTTCAACTACGTCTAGCTCGTCTTGTAGAGCCTGACCTTCTTCAACGGTGATAACACCATCACGGCCTACTTTTTCCATCGCTTCAGCAATGATGTTACCTACTGTCGCGTCAGAGTTTGCAGAGATAGTACCTACTTGTGCGATAGCTTTGGTATCTGCACATGGAACAGAAAGGCCTTTTAGCTCTTCAACTGCTGCAACAACGGCTTTGTCGATGCCGCGCTTAAGATCCATTGGGTTCATGCCAGCAGCAACGGCTTTTAGGCCTTCAGTGATGATAGCTTGAGCAAGAACAGTTGCCGTCGTCGTCCC

>10N.261.50.A12 TTCTAGAGTCGGAAGAAGTTCACGGATGTTAGACACTTTCTTGTCGATAAGAAGAATGAACGGGCTATCTAGATCAACAGAACCGGCTTCTTGGTTGTTGATGAAGTAAGGAGATAGGTAACCGCGGTCGAACTGCATACCTTCAACTACGTCTAGCTCATCTTGTAGAGCCTGACCTTCTTCAACCGTGATAACGCCATCACGACCTACTTTTTCCATCGCTTCAGCAATGATGTTACCCACTGTCGAATCAGAGTTCGCAGAGATAGTACCTACTTGCGCGATAGCTTTCGTGTCTGAACAAGGAACAGAAAGGTTCTTCAGCTCTTCAACAGCCGCGATAACCGCTTTGTCGATGCCGCGCTTAAGATCCATTGGGTTCATACCAGCAGCAACGGCTTTTAGGCCTTCAGCGATAATCGACTGAGCCAATACTGTTGCTGTCGTCGTCCC

>10N.261.50.G2 TTCTAGAGTCGGAAGAAGTTCACGGATGTTCGAAACTTTCTTGTCGATTAGAAGAATAAATGGGCTTTCTAGATCAACAGAACCTGCTTCTTGGTTGTTGATGAAGTAAGGAGACAGGTAACCGCGGTCGAACTGCATGCCTTCAACTACGTCTAGCTCGTCTTGCAGAGCCTGACCTTCTTCAACCGTGATTACGCCATCACGACCTACTTTTTCCATCGCTTCAGCAATGATGTTACCTACTGTCGAATCAGAGTTCGCAGAGATAGTACCTACTTGAGCGATAGCTTTCGTGTCTGAACAAGGAACAGAAAGGTTCTTCAGCTCTTCAACAGCCGCGATAACCGCTTTGTCGATGCCGCGCTTAAGATCCATTGGGTTCATTCCAGCAGCAACCGCTTTTAGACCTTCAGCGATAATAGACTGAGCCAATACTGTAGCTGTCGTCGTCCC

>10N.261.52.D5 TTCTAGAGTCGGAAGCAATTCACGGATGTTCGATACTTTCTTGTCGATAAGAAGAATGAATGGGCTTTCTAGATCAACAGAACCCGCTTCTTGGTTGTTGATGAAGTAAGGAGATAGGTAACCGCGGTCGAACTGCATACCTTCAACTACGTCTAGCTCGTCTTGTAGAGCCTGACCTTCTTCAACTGTGATAACGCCATCACGACCTACTTTTTCCATCGCTTCAGCAATGATGTTACCTACTGTCGAATCAGAGTTCGCAGAGATAGTACCTACTTGAGCGATAGCTTTCGTGTCTGAACAAGGAACAGAAAGGTTCTTCAGCTCTTCAACAGCCGCGATAACCGCTTTGTCGATGCCGCGCTTAAGATCCATTGGGTTCATGCCAGCAGCAACCGCTTTTAGGCCTTCAGCAATAATAGACTGAGCCAATACTGTTGCTGTCGTCGTCCC

>10N.261.52.D6 TTCTAGAGTCGGAAGCAATTCACGGATGTTCGATACTTTCTTGTCGATAAGAAGAATGAATGGGCTTTCTAGATCAACAGAACCCGCTTCTTGGTTGTTGATGAAGTAAGGAGATAGGTAACCGCGGTCGAACTGCATACCTTCAACTACGTCTAGCTCGTCTTGTAGAGCCTGACCTTCTTCAACTGTGATAACGCCATCACGACCTACTTTTTCCATCGCTTCAGCAATGATGTTACCTACTGTCGAATCAGAGTTCGCAGAGATAGTACCTACTTGAGCGATAGCTTTCGTGTCTGAACAAGGAACAGAAAGGTTCTTCAGCTCTTCAACAGCCGCGATAACCGCTTTGTCGATGCCGCGCTTAAGATCCATTGGGTTCATGCCAGCAGCAACCGCTTTTAGGCCTTCAGCAATAATAGACTGAGCCAATACTGTTGCTGTCGTCGTCCC

>10N.261.53.B8 TTCTAGAGTCGGAAGCAGTTCACGGATGTTCGATACTTTCTTGTCGATAAGAAGAATGAATGGGCTTTCTAGATCAACAGAACCCGCTTCTTGGTTGTTGATGAAGTAAGGAGATAGGTAACCGCGGTCGAACTGCATACCTTCCACTACGTCTAGCTCGTCTTGTAGAGCCTGACCTTCTTCAACTGTGATAACGCCATCACGACCTACTTTTTCCATCGCTTCAGCAATGATGTTACCTACTGTCGAATCAGAGTTCGCAGAGATAGTACCTACTTGAGCGATAGCTTTCGTGTCTGAACAAGGAACAGAAAGGTTCTTCAGCTCTTCAACAGCCGCGATAACCGCTTTGTCGATGCCGCGCTTAAGATCCATTGGGTTCATGCCAGCAGCAACCGCTTTTAGGCCTTCAGCAATAATAGACTGAGCCAATACTGTTGCTGTCGTCGTCCC

>10N.261.54.C1 TTCTAGAGTCGGAAGAAGTTCACGGATGTTCGACACTTTCTTGTCGATAAGAAGAATGAACGGGCTATCTAGATCAACAGAACCGGCTTCTTGGTTATTGATGAAGTAAGGAGATAGGTAACCGCGGTCGAACTGCATACCTTCAACTACGTCTAGCTCATCTTGTAGAGCCTGACCTTCTTCAACCGTGATAACGCCATCACGACCTACTTTTTCCATCGCTTCAGCAATGATGTTACCTACTGTCGAATCAGAGTTCGCAGAGATAGTACCTACTTGCGCGATAGCTTTCGTGTCTGAACAAGGAACAGAAAGGTTCTTCAGCTCTTCAACAGCCGCGATAACCGCTTTGTCGATGCCGCGCTTAAGATCCATTGGGTTCATGCCAGCAGCAACCGCTTTTAGGCCTTCAGCGATAATAGACTGAGCCAATACTGTTGCTGTCGTCGTCCC

>10N.261.54.E4 TTCTAGAGTCGGAAGAAGTTCACGGATGTTCGATACTTTCTTGTCGATAAGAAGAATGAATGGGCTTTCTAGATCAACAGAACCCGCTTCTTGGTTGTTGATGAAGTAAGGAGATAGGTAACCACGGTCGAACTGCATACCTTCAACTACGTCTAGCTCGTCTTGTAGAGCCTGACCTTCTTCAACTGTGATAACGCCATCACGACCTACTTTTTCCATCGCTTCAGCAATGATGTTACCTACTGTCGAATCAGAGTTCGCAGAGATAGTACCTACTTGAGCGATAGCTTTCGTGTCTGAACAAGGAACAGAAAGGTTCTTCAGCTCTTCAACAGCCGCGATAACCGCTTTGTCGATGCCGCGCTTAAGATCCATTGGGTTCATGCCAGCAGCAACCGCTTTTAGGCCTTCAGCGATAATAGACTGAGCCAATACTGTTGCTGTCGTCGTCCC

>10N.261.54.E7 TTCTAGAGTCGGAAGAAGTTCACGGATGTTCGATACTTTCTTGTCGATAAGAAGAATGAATGGGCTTTCTAGATCAACAGAACCCGCTTCTTGGTTGTTGATGAAGTAAGGAGATAGGTAACCGCGGTCGAACTGCATACCTTCCACTACGTCTAGCTCGTCTTGTAGAGCCTGACCTTCTTCAACCGTGATTACGCCATCACGACCTACTTTTTCCATCGCTTCAGCAATGATGTTACCTACTGTCGAATCAGAGTTCGCAGAGATAGTACCTACTTGAGCGATAGCTTTCGTGTCTGAACAAGGAACAGAAAGGTTCTTCAGCTCTTCAACAGCCGCGATAACCGCTTTGTCGATGCCGCGCTTAAGATCCATTGGGTTCATGCCAGCAGCAACCGCTTTTAGGCCTTCAGCGATAATAGACTGAGCCAATACTGTTGCTGTCGTCGTCCC

>10N.261.55.D10 TTCTAGAGTCGGAAGAAGTTCACGGATGTTCGAAACTTTCTTGTCGATTAGAAGAATGAATGGGCTTTCAAGATCAACAGAACCTGCTTCTTGGTTGTTGATGAAGTAAGGAGATAGGTAACCGCGGTCGAACTGCATACCTTCAACTACGTCTAGCTCGTCTTGCAGAGCCTGACCTTCTTCAACCGTGATTACGCCATCACGACCTACTTTTTCCATCGCTTCAGCAATGATGTTACCTACTGTCGAATCAGAGTTCGCAGAGATAGTACCGACTTGCGCGATAGCTTTCGTGTCTGAACAAGGAACAGAAAGGTTCTTCAGCTCTTCAACAGCCGCGATAACCGCTTTGTCGATGCCGCGCTTAAAATCCATTGGGTTCATGCCAGCCGCCACCGCTTTTAGGCCTTCAGCGATAATAGAATGAGCCAATTCTGTTGGTGTCGTCGTCCC

>10N.261.56.A5

TTCTAGCGTTGGAAGAAGCTCACGAATGTTTGAGATCTTCTTGTCTACTAGAAGAATGAATGGGTTTTCTAGATCAACACTGCCTGATTCTTGGTTGTTGATGAAGTAAGGAGACAGGTAACCGCGGTCGAACTGCATACCTTCTACTACGTCTAACTCGTCTTGTAGCGCCTGACCTTCTTCAACCGTGATAACGCCATCACGACCCACACGCTCCATTGCTTCTGCAATGATATTACCTACGCTTGCGTCTGAGTTTGCAGAGATAGTACCAACCTGCGCGATCGCTTTTGTGTCGTTACATTCAACAGACAGCTCTTTTAGTTGCTCAACTGCCGCGGCAACAGCTTTGTCGATACCACGCTTAAGATCCATTGGGTTCATGCCCGCTGCGACTGCTTTTAGACCTTCGTTAACGATAGATTGTGCTAGTACCGTTGCTGTCGTCGTCCC

>10N.261.56.C7 TTCTAGAGTCGGAAGAAGTTCACGGATGTTCGATACTTTCTTGTCGATAAGAAGAATGAATGGGCTTTCTAGATCAACAGAACCCGCTTCTTGGTTGTTGATGAAGTAAGGAGATAGGTAACCGCGGTCGAACTGCATACCTTCCACTACGTCTAGCTCGTCTTGTAGAGCCTGACCTTCTTCAACCGTGATTACGCCATCACGACCTACTTTTTCCATCGCTTCAGCAATGATGTTACCTACTGTCGAATCAGAGTTCGCAGAGATAGTACCTACTTGAGCGATAGCTTTCGTGTCTGAACAAGGAACAGAAAGGTTCTTCAGCTCTTCAACAGCCGCGATAACCGCTTTGTCGATGCCGCGCTTAAGATCCATTGGGTTCATGCCAGCAGCAACCGCTTTTAGGCCTTCAGCGATAATAGACTGAGCCAATACTGTTGCTGTCGTCGTCCC

>10N.261.56.C6 TTCTAGAGTCGGAAGCAATTCACGGATGTTCGATACTTTCTTGTCGATAAGAAGAATGAATGGGCTTTCTAGATCAACAGAACCCGCTTCTTGGTTGTTGATGAAGTAAGGAGATAGGTAACCGCGGTCGAACTGCATACCTTCAACTACGTCTAGCTCGTCTTGTAGAGCCTGACCTTCTTCAACTGTGATAACGCCATCACGACCTACTTTTTCCATCGCTTCAGCAATGATGTTACCTACTGTCGAATCAGAGTTCGCAGAGATAGTACCTACTTGAGCGATAGCTTTCGTGTCTGAACAAGGAACAGAAAGGTTCTTCAGCTCTTCAACAGCCGCGATAACCGCTTTGTCGATGCCGCGCTTAAGATCCATTGGGTTCATGCCAGCAGCAACCGCTTTTAGGCCTTCAGCAATAATAGACTGAGCCAATACTGTTGCTGTCGTCGTCCC

>10N.261.56.D9 TTCTAGAGTCGGAAGAAGTTCACGGATGTTCGATACTTTCTTGTCGATAAGAAGAATGAATGGGCTTTCTAGATCAACAGAACCCGCTTCTTGGTTGTTGATGAAGTAAGGAGATAGGTAACCGCGGTCGAACTGCATACCTTCCACTACGTCTAGCTCGTCTTGTAGAGCCTGACCTTCTTCAACCGTGATTACGCCATCACGACCTACTTTTTCCATCGCTTCAGCAATGATGTTACCTACTGTCGAATCAGAGTTCGCAGAGATAGTACCTACTTGAGCGATAGCTTTCGTGTCTGAACAAGGAACAGAAAGGTTCTTCAGCTCTTCAACAGCCGCGATAACCGCTTTGTCGATGCCGCGCTTAAGATCCATTGGGTTCATGCCAGCAGCAACCGCTTTTAGGCCTTCAGCGATAATAGACTGAGCCAATACTGTTGCTGTCGTCGTCCC

>10N.261.56.E1 TTCTAGAGTCGGAAGAAGCTCACGAATGTTCGAAATTTTCTTGTCGATAAGAAGAATGAATGGGCTTTCTAGATCAACAGAACCCGCTTCTTGGTTGTTGATGAAGTAAGGAGATAGGTAACCGCGGTCGAACTGCATACCTTCAACTACGTCTAGCTCGTCTTGTAGAGCCTGACCTTCTTCAACAGTGATAACGCCATCACGGCCTACTTTTTCCATCGCTTCAGCAATGATGTTACCCACTGTTGCGTCAGAGTTTGCAGAGATAGTACCTACTTGCGCGATAGCTTTTGTATCTGCACATGGAACCGAAAGGTTCTTCAGCTCTTCAACAGCCGCGATAACCGCTTTGTCGATGCCGCGCTTAAGATCCATTGGGTTCATACCCGCAGCAACCGCTTTTAGGCCTTCAGCGATAATCGACTGAGCCAATACTGTTGCTGTCGTCGTCCC

>10N.261.56.E4

TTCTAGCGTAGGAAGAAGCTCACGAATGTTTGAGATCTTCTTGTCTACTAGAAGAATGAATGGGTTTTCTAGATCAACACTGCCTGATTCTTGGTTGTTGATGAAGTAAGGAGACAGGTAACCGCGGTCGAACTGCATACCTTCTACTACGTCTAACTCGTCTTGTAGCGCCTGACCTTCTTCAACCGTGATAACGCCATCACGACCCACACGCTCCATTGCTTCTGCAATGATATTACCTACACTTGCATCTGAGTTTGCAGAGATAGTACCAACCTGCGCGATCGCTTTTGTGTCGTTACATTCAACAGAAAGCTCTTTTAGTTGCTCAACTGCCGCGGCAACAGCTTTGTCGATACCACGCTTAAGATCCATTGGGTTCATGCCCGCTGCGACTGCTTTTAGACCTTCGTTAACGATAGATTGTGCTAGTACCGTTGCTGTCGTCGTCCC

>10N.261.56.G4

TTCTAGCGTTGGAAGAAGCTCACGAATGTTTGAGATCTTCTTGTCTACCAGAAGAATGAATGGGTTTTCTAGATCAACACTGCCTGATTCTTGGTTGTTGATGAAGTAAGGAGACAGGTAACCGCGGTCGAACTGCATACCTTCTACTACGTCTAACTCGTCTTGTAGCGCCTGACCTTCTTCAACCGTGATAACGCCATCACGACCCACACGCTCCATTGCTTCTGCAATGATATTACCTACGCTTGCGTCTGAGTTTGCAGAGATAGTACCAACCTGCGCGATCGCTTTTGTGTCGTTACATTCAACAGAAAGCTCTTTTAGTTGCTCAACTGCCGCGGCAACAGCTTTGTCGATACCACGCTTAAGATCCATTGGGTTCATGCCCGCTGCGACTGCTTTTAGACCTTCGTTAACGATAGATTGTGCTAGTACCGTTGCTGTCGTCGTCCC

>10N.286.45.B6 TTCTAGAGTCGGAAGAAGTTCACGGATATTTGAAACTTTCTTGTCGATTAGAAGGATGAATGGGCTTTCTAGATCAACAGAACCTGCTTCTTGGTTGTTGATGAAGTAAGGAGATAGGTAACCGCGGTCAAACTGCATACCTTCAACTACGTCTAGCTCGTCTTGTAGAGCCTGACCTTCTTCAACGGTGATAACACCATCACGGCCTACTTTTTCCATCGCTTCAGCAATGATGTTACCTACTGTCGCGTCAGAGTTTGCAGAGATAGTACCTACTTGTGCGATAGCTTTGGTATCTGCACATGGAACAGAAAGGCCTTTTAGCTCTTCAACTGCTGCAACAACGGCTTTGTCGATGCCGCGCTTAAGATCCATTGGGTTCATGCCAGCAGCAACGGCTTTTAGGCCTTCAGTGATGATAGCTTGAGCAAGAACAGTTGCCGTCGTCGTCCC

>10N.286.45.B8 TTCTAGAGTCGGAAGAAGTTCACGGATGTTCGACACTTTCTTGTCGATAAGAAGAATGAATGGGCTTTCTAGATCAACAGAACCTGCTTCTTGGTTGTTGATGAAGTAAGGAGATAGGTAACCGCGGTCGAACTGCATACCTTCCACTACGTCTAGCTCGTCTTGCAGAGCCTGACCTTCTTCAACCGTGATTACGCCATCACGACCTACTTTTTCCATCGCTTCAGCAATGATGTTACCTACTGTCGAATCAGAGTTCGCAGAGATAGTACCTACTTGAGCGATAGCTTTAGTGTCTGAACAAGGAACAGAAAGGTTCTTCAGCTCTTCAACAGCCGCGATAACCGCTTTGTCGATGCCGCGCTTAAGATCCATTGGGTTCATGCCCGCAGCAACCGCTTTGAGGCCTTCAGCAATAATAGATTGAGCCAATACTGTAGCTGTCGTCGTCCC

>10N.286.45.F3 TTCTAGAGTCGGAAGAAGTTCACGGATATTTGAAACTTTCTTGTCGATTAGAAGGATGAATGGGCTTTCTAGATCAACAGAACCTGCTTCTTGGTTGTTGATGAAGTAAGGAGATAGGTAACCGCGGTCAAACTGCATACCTTCAACTACGTCTAGCTCGTCTTGTAGAGCCTGACCTTCTTCAACGGTGATAACACCATCACGGCCTACTTTTTCCATCGCTTCAGCAATGATGTTACCTACTGTCGCGTCAGAGTTTGCAGAGATAGTACCTACTTGTGCGATAGCTTTGGTATCTGCACATGGAACAGAAAGGCCTTTTAGCTCTTCAACTGCTGCAACAACGGCTTTGTCGATGCCGCGCTTAAGATCCATTGGGTTCATGCCAGCAGCAACGGCTTTTAGGCCTTCAGTGATGATAGCTTGAGCAAGAACAGTTGCCGTCGTCGTCCC

>10N.286.46.A6 TTCTAGAGTCGGAAGAAGTTCACGGATATTTGAAACTTTCTTGTCGATTAGAAGGATGAATGGGCTTTCTAGATCAACAGAACCTGCTTCTTGGTTGTTGATGAAGTAAGGAGATAGGTAACCGCGGTCAAACTGCATACCTTCAACTACGTCTAGCTCGTCTTGTAGAGCCTGACCTTCTTCAACGGTGATAACACCATCACGGCCTACTTTTTCCATCGCTTCAGCAATGATGTTACCTACTGTCGCGTCAGAGTTTGCAGAGATAGTACCTACTTGTGCGATAGCTTTGGTATCTGCACATGGAACAGAAAGGCCTTTTAGCTCTTCAACTGCTGCAACAACGGCTTTGTCGATGCCGCGCTTAAGATCCATTGGGTTCATGCCAGCAGCAACGGCTTTTAGGCCTTCAGTGATGATAGCTTGAGCAAGAACAGTTGCCGTCGTCGTCCC

>10N.286.46.E3 TTCTAGAGTCGGAAGAAGTTCACGGATGTTCGAAACTTTCTTGTCGATAAGCAGAATGAATGGGCTTTCTAGATCAACAGAACCCGCTTCTTGGTTATTGATGAAGTAAGGAGATAGGTAACCGCGATCGAACTGCATACCTTCAACTACGTCTAGCTCGTCTTGTAGAGCCTGGCCTTCTTCAACTGTGATTACGCCATCACGGCCTACTTTTTCCATCGCTTCAGCAATGATGTTACCTACTGTCGAATCCGAGTTCGCAGAGATAGTACCTACTTGAGCGATAGCTTTAGTATCAGAACATGGAACAGAAAGGCCTTTAAGCTCTTCAACTGCTGCGATAACCGCTTTGTCGATGCCGCGCTTAAGATCCATTGGGTTCATGCCAGCAGCAACGGCTTTTAGGCCTTCAGTGATGATTGCTTGTGCAAGAACTGTCGCCGTCGTCGTCCC

>10N.286.46.E4 TTCTAGAGTCGGAAGAAGTTCACGGATGTTCGAAACTTTCTTGTCGATAAGCAGAATGAATGGGCTTTCTAGATCAACAGAACCCGCTTCTTGGTTATTGATGAAGTAAGGAGATAGGTAACCGCGATCGAACTGCATACCTTCAACTACGTCTAGCTCGTCTTGTAGAGCCTGGCCTTCTTCAACTGTGATTACGCCATCACGGCCTACTTTTTCCATCGCTTCAGCAATGATGTTACCTACTGTCGAATCCGAGTTCGCAGAGATAGTACCTACTTGAGCGATAGCTTTAGTATCAGAACATGGAACAGAAAGGCCTTTAAGCTCTTCAACTGCTGCGATAACCGCTTTGTCGATGCCGCGCTTAAGATCCATTGGGTTCATGCCAGCAGCAACGGCTTTTAGGCCTTCAGTGATGATTGCTTGTGCAAGAACTGTCGCCGTCGTCGTCCC

>10N.286.46.F3 TTCTAGAGTCGGAAGAAGTTCACGGATGTTCGAAACTTTCTTGTCGATAAGCAGAATGAATGGGCTTTCTAGATCAACAGAACCCGCTTCTTGGTTATTGATGAAGTAAGGAGATAGGTAACCGCGATCGAACTGCATACCTTCAACTACGTCTAGCTCGTCTTGTAGAGCCTGGCCTTCTTCAACTGTGATTACGCCATCACGGCCTACTTTTTCCATCGCTTCAGCAATGATGTTACCTACTGTCGAATCCGAGTTCGCAGAGATAGTACCTACTTGAGCGATAGCTTTAGTATCAGAACATGGAACAGAAAGGCCTTTAAGCTCTTCAACTGCTGCGATAACCGCTTTGTCGATGCCGCGCTTAAGATCCATTGGGTTCATGCCAGCAGCAACGGCTTTTAGGCCTTCAGTGATGATTGCTTGTGCAAGAACTGTCGCCGTCGTCGTCCC

>10N.286.46.F4 TTCTAGAGTCGGAAGAAGTTCACGGATGTTCGAAACTTTCTTGTCGATAAGCAGAATGAATGGGCTTTCTAGATCAACAGAACCCGCTTCTTGGTTATTGATGAAGTAAGGAGATAGGTAACCGCGATCGAACTGCATACCTTCAACTACGTCTAGCTCGTCTTGTAGAGCCTGGCCTTCTTCAACTGTGATTACGCCATCACGGCCTACTTTTTCCATCGCTTCAGCAATGATGTTACCTACTGTCGAATCCGAGTTCGCAGAGATAGTACCTACTTGAGCGATAGCTTTAGTATCAGAACATGGAACAGAAAGGCCTTTAAGCTCTTCAACTGCTGCGATAACCGCTTTGTCGATGCCGCGCTTAAGATCCATTGGGTTCATGCCAGCAGCAACGGCTTTTAGGCCTTCAGTGATGATTGCTTGTGCAAGAACTGTCGCCGTCGTCGTCCC

>10N.286.47.A9 TTCTAGAGTCGGAAGAAGTTCACGGATGTTCGACACTTTCTTGTCAATAAGAAGAATGAACGGGCTATCTAGATCAACAGAACCGGCTTCTTGGTTGTTGATGAAGTAAGGAGATAGGTAACCGCGGTCGAACTGCATACCTTCAACTACGTCTAGCTCGTCTTGCAGAGCCTGACCTTCTTCAACCGTGATTACACCATCACGACCTACTTTTTCCATCGCTTCAGCAATGATGTTACCTACTGTTGAATCAGAGTTCGCAGAGATAGTACCTACTTGCGCGATAGCTTTCGTGTCTGAACAAGGAACAGAAAGGTTCTTCAGCTCTTCAACAGCCGCGATAACCGCTTTGTCGATGCCGCGCTTAAGATCCATTGGGTTCATGCCAGCAGCAACCGCTTTTAGGCCTTCAGCGATAATAGACTGAGCCAATACTGTTGCTGTCGTCGTCCC

>10N.286.47.B9

TTCAAGTGCAGGAAGCAGTTCGCGAATGTTAGAAATTTTCTTATCAACCAATAGGATGAATGGGCTATCTAGATCAACACTGCCCGCTTCTTGGTTATTAATGAAGTAAGGAGATAGGTAACCACGATCAAACTGCATGCCTTCCACGACATCGAGTTCATCTTGTAGTGCTTGGCCTTCTTCAACAGTGATAACGCCATCTCGGCCCACTTTTTCCATCGCTTCAGCAATGATGTTACCCACGCTTGAATCCGAGTTCGCAGAAATAGTACCCACTTGCGCAATCGCTTTAGTGTCTGAACAAGGTACTGAAAGCTCTTTCAACGCTTCAACCGCCGCCACGACAGCTTTGTCGATGCCACGCTTAAGATCCATTGGGTTCATACCCGCTGCTACAGCTTTCAAGCCTTCAGTGATGATCGCTTGCGCTAATACCGTTGCGGTCGTCGTCCC

>10N.286.47.C2 TTCTAGAGTCGGAAGAAGCTCACGAATGTTCGAAATCTTCTTGTCGATAAGAAGAATGAATGGGCTTTCTAGATCAACAGAACCCGCTTCTTGGTTGTTGATGAAGTAAGGAGATAGGTAACCGCGGTCGAACTGCATACCTTCAACTACGTCTAGCTCGTCTTGTAGAGCCTGACCTTCTTCAACAGTGATAACGCCATCACGGCCTACTTTTTCCATCGCTTCAGCAATGATGTTACCCACTGTTGCGTCAGAGTTTGCAGAGATAGTACCTACTTGCGCGATAGCTTTCGTATCTGCACATGGAACAGAAAGGTTCTTCAGCTCTTCAACAGCCGCGATAACCGCTTTGTCGATGCCGCGCTTAAGATCCATTGGGTTCATACCCGCAGCAACCGCTTTTAGGCCTTCAGCGATAATCGACTGAGCCAATACTGTTGCTGTCGTCGTCCC

>10N.286.47.D3

TTCAAGTGCAGGAAGCAGTTCGCGAATGTTAGAAATTTTCTTATCAACCAATAGGATGAATGGGCTATCTAGATCAACACTGCCCGCTTCTTGGTTATTAATGAAGTAAGGAGATAGGTAACCACGATCAAACTGCATGCCTTCCACGACATCGAGTTCATCTTGTAGTGCTTGGCCTTCTTCAACAGTGATAACGCCATCTCGGCCCACTTTTTCCATCGCTTCAGCAATGATGTTACCCACGCTTGAATCCGAGTTCGCAGAAATAGTACCCACTTGCGCAATCGCTTTAGTGTCTGAACAAGGTACTGAAAGCTCTTTCAACGCTTCAACCGCCGCCACGACAGCTTTGTCGATGCCACGCTTAAGATCCATTGGGTTCATACCCGCTGCTACAGCTTTCAAGCCTTCAGTGATGATCGCTTGCGCTAATACCGTTGCGGTCGTCGTCCC

>10N.286.47.D4 TTCAAGTGCAGGAAGCAGTTCGCGAATGTTAGAAATTTTCTTATCAACCAATAGGATGAATGGGCTATCTAGATCAACACTGCCCGCTTCTTGGTTATTAATGAAGTAAGGAGATAGGTAACCACGATCAAACTGCATGCCTTCCACGACATCGAGTTCATCTTGTAGTGCTTGGCCTTCTTCAACAGTGATAACGCCATCTCGGCCCACTTTTTCCATCGCTTCAGCAATGATGTTACCCACGCTTGAATCCGAGTTCGCAGAAATAGTACCCACTTGCGCAATCGCTTTAGTGTCTGAACAAGGTACTGAAAGCTCTTTCAACGCTTCAACCGCCGCCACGACAGCTTTGTCGATGCCACGCTTAAGATCCATTGGGTTCATACCCGCTGCTACAGCTTTCAAGCCTTCAGTGATGATCGCTTGCGCTAATACCGTTGCGGTCGTCGTCCC

>10N.286.47.F2

TTCAAGTGCAGGAAGCAGTTCGCGAATGTTAGAAATTTTCTTATCAACCAATAGGATGAATGGGCTATCTAGATCAACACTGCCCGCTTCTTGGTTATTAATGAAGTAAGGAGATAGGTAACCACGATCAAACTGCATGCCTTCCACGACATCGAGTTCATCTTGTAGTGCTTGGCCTTCTTCAACAGTGATAACGCCATCTCGGCCCACTTTTTCCATCGCTTCAGCAATGATGTTACCCACGCTTGAATCCGAGTTCGCAGAAATAGTACCCACTTGCGCAATCGCTTTAGTGTCTGAACAAGGTACTGAAAGCTCTTTCAACGCTTCAACCGCCGCCACGACAGCTTTGTCGATGCCACGCTTAAGATCCATTGGGTTCATACCCGCTGCTACAGCTTTCAAGCCTTCAGTGATGATCGCTTGCGCTAATACCGTTGCGGTCGTCGTCCC

>10N.286.48.D5 TTCTAGAGTCGGAAGAAGTTCACGGATGTTCGAAACTTTCTTGTCGATAAGAAGAATGAATGGGCTTTCTAGATCAACAGAACCTGCTTCTTGGTTGTTGATGAAGTAAGGAGACAGGTAACCGCGATCGAACTGCATACCTTCAACTACGTCTAGCTCGTCTTGTAGAGCCTGACCTTCTTCAACTGTGATTACGCCATCACGACCTACTTTTTCCATCGCTTCAGCAATGATGTTACCTACTGTCGAATCAGAGTTCGCAGAGATAGTACCTACTTGCGCGATAGCTTTCGTGTCTGAACAAGGAACAGAAAGGTTCTTCAGCTCTTCAACAGCCGCGATAACCGCTTTGTCGATGCCACGCTTAAGATCCATTGGGTTCATGCCAGCAGCAACTGCTTTTAGGCCTTCAGCGATAATAGACTGAGCCAATACTGTAGCTGTCGTCGTCCC

>10N.286.48.E5 TTCTAGAGTCGGAAGAAGTTCACGGATGTTCGACACTTTCTTGTCGATAAGAAGAATGAACGGGCTATCTAGATCAACAGAACCGGCTTCTTGGTTGTTGATGAAGTAAGGAGATAGGTAACCGCGGTCGAACTGCATACCTTCAACTACGTCTAACTCGTCTTGCAGAGCCTGACCTTCTTCAACCGTGATAACGCCATCACGACCTACTTTTTCCATCGCTTCAGCAATGATGTTACCTACTGTCGAATCAGAGTTCGCAGAGATAGTACCTACTTGCGCGATAGCTTTCGTGTCTGAACAAGGAACAGAAAGGTTCTTCAGCTCTTCAACAGCCGCGATAACCGCTTTGTCGATGCCGCGCTTAAGATCCATTGGGTTCATACCAGCAGCAACCGCTTTTAGGCCTTCAGCGATAATAGACTGAGCCAATACTGTTGCTGTCGTCGTCCC

>10N.286.49.C6 TTCTAGAGTCGGAAGCAGTTCACGGATGTTCGATACTTTCTTGTCGATAAGAAGAATGAATGGGCTTTCTAGATCAACAGAACCCGCTTCTTGGTTGTTGATGAAGTAAGGAGATAGGTAACCGCGGTCGAACTGCATACCTTCCACTACGTCTAGCTCGTCTTGTAGAGCCTGACCTTCTTCAACTGTGATAACGCCATCACGACCTACTTTTTCCATCGCTTCAGCAATGATGTTACCTACTGTCGAATCAGAGTTCGCAGAGATAGTACCTACTTGAGCGATAGCTTTCGTGTCTGAACAAGGAACAGAAAGGTTCTTCAGCTCTTCAACAGCCGCGATAACCGCTTTGTCGATGCCGCGCTTAAGATCCATTGGGTTCATGCCAGCAGCAACCGCTTTTAGGCCTTCAGCAATAATAGACTGAGCCAATACTGTTGCTGTCGTCGTCCC

>10N.286.49.D1 TTCTAGAGTCGGAAGAAGTTCACGGATATTCGAAACTTTCTTGTCGATAAGAAGAATGAATGGGCTTTCTAAATCAACAGAACCTGCTTCTTGGTTGTTGATGAAGTAAGGAGACAGGTAACCGCGGTCGAACTGCATACCTTCAACTACGTCTAGCTCGTCTTGCAGAGCCTGACCTTCTTCAACCGTGATTACGCCATCACGACCTACTTTTTCCATCGCTTCAGCAATGATGTTACCTACTGTCGAATCAGAGTTCGCAGAGATAGTACCTACTTGCGCGATAGCTTTCGTGTCTGAACAAGGAACAGAAAGGTTCTTCAACTCTTCAACAGCCGCGATAACCGCTTTGTCGATGCCGCGCTTAAGATCCATTGGGTTCATGCCAGCAGCAACCGCTTTTAGGCCTTCTGCGATAATAGACTGAGCCAATACTGTTGCTGTCGTCGTCCC

>10N.286.50.H3 TTCTAGAGTCGGAAGAAGTTCACGGATGTTCGACACTTTCTTGTCGATAAGAAGAATGAACGGGCTATCTAGATCAACAGAACCGGCTTCTTGGTTGTTGATGAAGTAAGGAGATAGGTAACCGCGGTCGAACTGCATACCTTCAACTACGTCTAACTCGTCTTGCAGAGCCTGACCTTCTTCAACCGTGATAACGCCATCACGACCTACTTTTTCCATCGCTTCAGCAATGATGTTACCTACTGTCGAATCAGAGTTCGCAGAGATAGTACCTACTTGCGCGATAGCTTTTGTGTCTGAACAAGGAACAGAAAGGTTCTTCAGCTCTTCAACAGCCGCGATAACCGCTTTGTCGATGCCGCGCTTAAGATCCATTGGGTTCATGCCAGCAGCAACCGCTTTTAGGCCTTCAGCGATAATAGACTGAGCCAATACTGTTGCTGTCGTCGTCCC

>10N.286.52.B3 TTCTAGAGTCGGAAGAAGTTCACGGATGTTCGACACTTTCTTGTCAATAAGAAGAATGAACGGGCTATCTAGATCAACAGAACCTGCTTCTTGGTTGTTGATGAAGTAAGGAGATAGGTAACCGCGGTCGAACTGCATACCTTCAACTACGTCTAGCTCGTCTTGCAGAGCCTGACCTTCTTCAACTGTGATAACGCCATCACGACCTACTTTTTCCATCGCTTCAGCAATGATGTTACCTACTGTCGAATCAGAGTTCGCAGAGATAGTACCTACTTGCGCGATAGCTTTCGTGTCTGAACAAGGAACAGAAAGGTTCTTCAGCTCTTCAACAGCCGCGATAACCGCTTTGTCGATGCCGCGCTTAAGATCCATTGGGTTCATGCCAGCAGCAACCGCTTTTAGGCCTTCAGCGATAATAGACTGAGCCAATACTGTTGCTGTCGTCGTCCC

>10N.286.52.B6 TTCTAGAGTCGGAAGAAGTTCACGGATGTTCGACACTTTCTTGTCAATAAGAAGAATGAACGGGCTATCTAGATCAACAGAACCTGCTTCTTGGTTGTTGATGAAGTAAGGAGATAGGTAACCGCGGTCGAACTGCATACCTTCAACTACGTCTAGCTCGTCTTGCAGAGCCTGACCTTCTTCAACTGTGATAACGCCATCACGACCTACTTTTTCCATCGCTTCAGCAATGATGTTACCTACTGTCGAATCAGAGTTCGCAGAGATAGTACCTACTTGCGCGATAGCTTTCGTGTCTGAACAAGGAACAGAAAGGTTCTTCAGCTCTTCAACAGCCGCGATAACCGCTTTGTCGATGCCGCGCTTAAGATCCATTGGGTTCATGCCAGCAGCAACCGCTTTTAGGCCTTCAGCGATAATAGACTGAGCCAATACTGTTGCTGTCGTCGTCCC

>10N.286.52.C6

TTCTAGAGTCGGAAGAAGTTCACGGATGTTCGACACTTTCTTGTCAATAAGAAGAATGAACGGGCTATCTAGATCAACAGAACCTGCTTCTTGGTTGTTGATGAAGTAAGGAGATAGGTAACCGCGGTCGAACTGCATACCTTCAACTACGTCTAGCTCGTCTTGCAGAGCCTGACCTTCTTCAACTGTGATAACGCCATCACGACCTACTTTTTCCATCGCTTCAGCAATGATGTTACCTACTGTCGAATCAGAGTTCGCAGAGATAGTACCTACTTGCGCGATAGCTTTCGTGTCTGAACAAGGAACAGAAAGGTTCTTCAGCTCTTCAACAGCCGCGATAACCGCTTTGTCGATGCCGCGCTTAAGATCCATTGGGTTCATGCCAGCAGCAACCGCTTTTAGGCCTTCAGCGATAATAGACTGAGCCAATACTGTTGCTGTCGTCGTCCC

>10N.286.52.D6

TTCTAGAGTCGGAAGAAGTTCACGGATGTTCGACACTTTCTTGTCAATAAGAAGAATGAACGGGCTATCTAGATCAACAGAACCTGCTTCTTGGTTGTTGATGAAGTAAGGAGATAGGTAACCGCGGTCGAACTGCATACCTTCAACTACGTCTAGCTCGTCTTGCAGAGCCTGACCTTCTTCAACTGTGATAACGCCATCACGACCTACTTTTTCCATCGCTTCAGCAATGATGTTACCTACTGTCGAATCAGAGTTCGCAGAGATAGTACCTACTTGCGCGATAGCTTTCGTGTCTGAACAAGGAACAGAAAGGTTCTTCAGCTCTTCAACAGCCGCGATAACCGCTTTGTCGATGCCGCGCTTAAGATCCATTGGGTTCATGCCAGCAGCAACCGCTTTTAGGCCTTCAGCGATAATAGACTGAGCCAATACTGTTGCTGTCGTCGTCCC

>10N.286.55.C1

TTTTAGAGTCGGAAGAAGTTCACGGATGTTCGACACTTTCTTGTCGATTAGAAGAATAAATGGGCTTTCTAGATCAACAGAACCTGCTTCTTGGTTGTTGATGAAGTAAGGAGATAGGTAACCGCGGTCGAACTGCATACCTTCCACTACGTCTAGCTCGTCTTGCAGAGCCTGACCTTCTTCAACCGTGATTACGCCATCACGACCTACTTTTTCCATCGCTTCAGCAATGATGTTACCTACTGTCGAATCAGAGTTCGCAGAGATAGTACCTACTTGAGCGATAGCTTTCGTGTCTGAACAAGGAACAGAAAGGTTCTTCAGCTCTTCAACAGCCGCGATAACCGCTTTGTCGATGCCGCGCTTAAGATCCATTGGGTTCATGCCCGCAGCAACCGCTTTTAGGCCTTCAGCGATAATAGACTGAGCCAATACTGTTGCTGTCGTCGTCCC

>10N.286.55.C2 TTCTAGAGTCGGAAGAAGTTCACGGATGTTCGACACTTTCTTGTCGATTAGAAGAATAAATGGGCTTTCTAGATCAACAGAACCTGCTTCTTGGTTGTTGATGAAGTAAGGAGATAGGTAACCGCGGTCGAACTGCATACCTTCCACTACGTCTAGCTCGTCTTGCAGAGCCTGACCTTCTTCAACCGTGATTACGCCATCACGACCTACTTTTTCCATCGCTTCAGCAATGATGTTACCTACTGTCGAATCAGAGTTCGCAGAGATAGTACCTACTTGAGCGATAGCTTTCGTGTCTGAACAAGGAACAGAAAGGTTCTTCAGCTCTTCAACAGCCGCGATAACCGCTTTGTCGATGCCGCGCTTAAGATCCATTGGGTTCATGCCCGCAGCAACCGCTTTTAGGCCTTCAGCGATAATAGACTGAGCCAATACTGTTGCTGTCGTCGTCCC

>10N.286.55.C3 TTCTAGAGTCGGAAGAAGCTCACGAATGTTCGAAATTTTCTTGTCGATAAGAAGAATGAATGGGCTTTCTAGATCAACAGAACCCGCTTCTTGGTTGTTGATGAAGTAAGGAGATAGGTAACCGCGGTCGAACTGCATACCTTCAACTACGTCTAGCTCGTCTTGTAGAGCCTGACCTTCTTCAACAGTGATAACGCCATCACGGCCTACTTTTTCCATCGCTTCAGCAATGATGTTACCCACTGTTGCGTCAGAGTTTGCAGAGATAGTACCTACTTGCGCGATAGCTTTCGTATCTGCACATGGAACAGAAAGGTTCTTCAGTTCTTCTACAGCCGCGATAACCGCTTTGTCGATACCGCGCTTAAGATCCATTGGGTTCATACCCGCAGCAACCGCTTTTAGGCCTTCAGCGATAATCGACTGAGCCAATACTGTTGCTGTCGTCGTCCC

>10N.286.55.C8 TTCTAGAGTCGGAAGAAGCTCACGAATGTTCGAAATTTTCTTGTCGATAAGAAGAATGAATGGGCTTTCTAGATCAACAGAACCCGCTTCTTGGTTGTTGATGAAGTAAGGAGATAGGTAACCGCGGTCGAACTGCATACCTTCAACTACGTCTAGCTCGTCTTGTAGAGCCTGACCTTCTTCAACAGTGATAACGCCATCACGGCCTACTTTTTCCATCGCTTCAGCAATGATGTTACCCACTGTAGCGTCAGAGTTTGCAGAGATAGTACCTACTTGCGCGATAGCTTTTGTATCTGCACATGGAACAGAAAGGTTCTTCAGTTCTTCTACAGCCGCGATAACCGCTTTGTCGATACCGCGCTTAAGATCCATTGGGTTCATACCCGCAGCAACCGCTTTTAGGCCTTCAGCGATAATCGACTGAGCCAATACTGTTGCTGTCGTCGTCCC

>10N.286.55.B4 TTCTAGAGTCGGAAGAAGCTCACGAATGTTCGAAATCTTCTTGTCGATAAGAAGAATGAATGGGCTTTCTAGATCAACAGAACCCGCTTCTTGGTTGTTGATGAAGTAAGGAGATAGGTAACCGCGGTCGAACTGCATACCTTCAACTACGTCTAGCTCGTCTTGTAGAGCCTGACCTTCTTCAACAGTGATAACGCCATCACGGCCGACTTTTTCCATCGCTTCAGCAATGATGTTACCCACTGTAGCGTCAGAGTTTGCAGAGATAGTACCGACTTGCGCGATAGCTTTCGTATCTGCACATGGAACAGAAAGGTTCTTCAGTTCTTCTACAGCCGCGATAACCGCTTTGTCGATGCCGCGCTTAAGATCCATTGGGTTCATACCCGCAGCAACTGCTTTTAGGCCTTCAGCGATAATCGACTGAGCCAATACTGTTGCTGTCGTCGTCCC

>10N.286.55.D3 TTCTAGAGTCGGAAGAAGTTCACGGATGTTCGAAACTTTCTTGTCGATTAGAAGAATGAATGGGCTTTCTAGATCAACAGAACCGGCTTCTTGGTTGTTGATGAAGTAAGGAGATAGGTAACCGCGGTCGAACTGCATACCTTCAACTACGTCTAGCTCATCTTGTAGAGCCTGACCTTCTTCAACCGTGATAACGCCATCACGACCTACTTTTTCCATCGCTTCAGCAATGATGTTACCCACTGTCGAATCAGAGTTCGCAGAGATAGTACCTACTTGAGCGATAGCTTTCGTGTCTGAACAAGGAACAGATAGGTTCTTCAGCTCTTCAACAGCCGCGATAACCGCTTTGTCGATGCCGCGCTTAAGATCCATTGGATTCATGCCAGCAGCAACGGCTTTTAGGCCTTCAGCGATAATAGACTGAGCCAATACTGTTGCTGTCGTCGTCCC
